# Quantitative Proteome Profiling of *C. burnetii* under Tetracycline Stress Conditions

**DOI:** 10.1371/journal.pone.0033599

**Published:** 2012-03-16

**Authors:** Iosif Vranakis, Pieter-Jan De Bock, Anastasia Papadioti, Yannis Tselentis, Kris Gevaert, Georgios Tsiotis, Anna Psaroulaki

**Affiliations:** 1 Department of Clinical Bacteriology, Parasitology, Zoonoses, and Geographical Medicine, Medical School, University of Crete, Heraklion, Greece; 2 Division of Biochemistry, Department of Chemistry, University of Crete, Voutes, Greece; 3 Department of Medical Protein Research, VIB, Ghent, Belgium; 4 Department of Biochemistry, Ghent University, Ghent, Belgium; Tulane University School of Medicine, United States of America

## Abstract

The recommended antibiotic regimen against *Coxiella burnetii*, the etiological agent of Q fever, is based on a semi-synthetic, second-generation tetracycline, doxycycline. Here, we report on the comparison of the proteomes of a *C. burnetii* reference strain either cultured under control conditions or under tetracycline stress conditions. Using the MS-driven combined fractional diagonal chromatography proteomics technique, out of the 531 proteins identified, 5 and 19 proteins were found significantly up- and down-regulated respectively, under tetracycline stress. Although the predicted cellular functions of these regulated proteins did not point to known tetracycline resistance mechanisms, our data clearly reveal the plasticity of the proteome of *C. burnetii* to battle tetracycline stress. Finally, we raise several plausible hypotheses that could further lead to more focused experiments on studying tetracycline resistance in *C. burnetii* and thus reduced treatment failures of Q fever.

## Introduction

*Coxiella burnetii*, until recently considered to be an obligatory intracellular bacterium [1], [2], is the causative agent of Q fever. Since its first documentation in abattoir workers in Queensland (Australia), where the need for diagnosis based on vague febrile symptoms led to an illness named Query (Q) fever [3], the clinical manifestations observed are numerous. In fact, the main characteristic of Q fever is its clinical polymorphism. Although *C. burnetii* infection can result in outcomes ranging from asymptomatic seroconversion to death, the most typical clinical manifestations of acute Q fever are fever (91%), severe headaches (51%), myalgias (37%), arthralgias (27%) and cough (34%) [4]. Chronic Q fever can develop many months or years after infection, manifesting itself in the majority of cases as endocarditis [5] and more rarely as osteomyelitis, osteoarthritis, hepatitis, and other manifestations [6].

Treatment of both acute as well as chronic Q fever is based on doxycycline, a semi-synthetic and second-generation tetracycline invented and clinically developed in the early 1960s [7] [8]. The recommended regimen for treating acute Q fever is doxycycline for two weeks, with fluoroquinolones suggested as reliable alternatives, in particular for patients with Q fever meningoencephalitis, due to their efficient penetration into the cerebrospinal fluid [8]. Further, cotrimoxazole and rifampin can be used in cases of tetracycline contraindication [4].

Doxycycline in combination with hydroxychloroquine is the main therapy for treating patients with chronic Q fever. However, the optimal duration of therapy for chronic Q fever is unknown and ranges from 18 months to life-long antibiotic administration [9]. Of interest is that *C. burnetii* strains resistant to doxycycline (MIC of 8 mg/mL) have been isolated from patients with Q fever endocarditis [10]. Furthermore, *C. burnetii* was recovered from cardiac valve tissue removed from a patient with Q fever endocarditis despite 4 years of antibiotic therapy with tetracycline [11].

Tetracyclines are broad-spectrum agents and exhibit antibiotic activity against a wide range of microorganisms. Their favorable antimicrobial properties and the absence of major, adverse side-effects have led to their extensive use in treating infected humans and animals. Tetracyclines inhibit bacterial protein synthesis by preventing the association of aminoacyl-tRNA with the bacterial ribosome [12]. Indeed, binding of tetracyclines sterically blocks aminoacyl-tRNA binding and, as a result, inhibits protein synthesis [13]. Tetracyclines binding to ribosomes is reversible, hence explaining their bacteriostatic, rather than bactericidal effects [12].

Resistance to tetracyclines mainly occurs through five mechanisms: production of ribosomal protection proteins (RPPs), active efflux of tetracycline from the cell, decreased drug permeability, mutation of the antibiotic target and their enzymatic degradation. Of note is that the first two mechanisms currently predominate in clinical settings [14].

Here, we compared the proteomes of the reference strain *C. burnetii* (CbuG_Q212) either propagated in the presence of doxycycline or propagated without any antibiotic present. The CbuG_Q212 strain was particularly chosen since it is considered as a prototype *C. burnetii* chronic disease isolate [15]. Chances are that a chronic disease isolate may remain under tetracycline stress for prolonged periods of time compared to an acute disease reference strain such as Nine Mile in *in vivo* conditions and might have increased possibilities to develop drug resistance. The COFRADIC proteomics technology (COmbined FRActional DIagonal Chromatography) was used here, which is a so-called gel-free proteomics technology based on the principle of diagonal chromatography [16]. Since COFRADIC depends on mass spectrometry (MS) for protein identification and quantification, typically a more comprehensive proteome coverage is achieved as compared to gel-based proteomics methods [16]. The major aim of the current study was to provide further insights into possible adaptations of the bacterial proteome under antibiotic stress conditions. The ultimate goal we envision is to identify the molecular mechanisms *Coxiella burnetii* can implement to resist protein synthesis inhibition by tetracyclines..

## Results

### Protein identification

**Figure 1:**
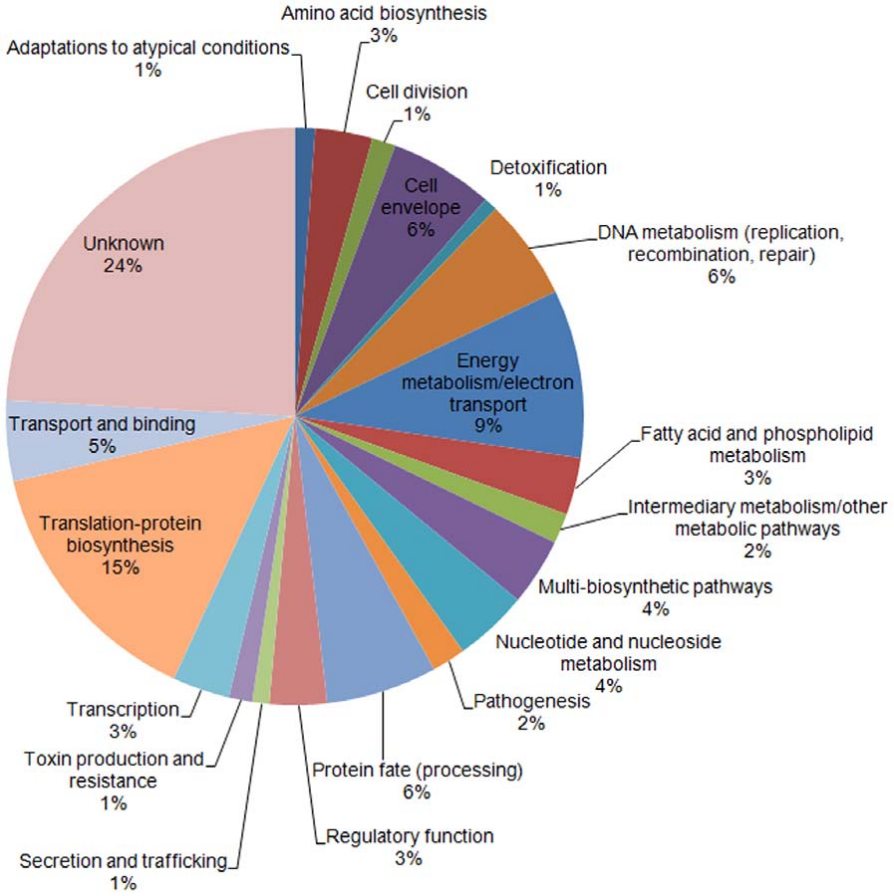
Classification of the 531 identified *C. burnetii* proteins according to their cellular function. These 531 proteins were identified in both samples; i. e., *C. burnetii* cultured in presence of tetracycline and *C. burnetii* cultured with no antibiotic present.

The COFRADIC technology used here enriches for methionine-containing peptides out of protease-digested proteomes with the aim to reduce the sample complexity before the actual LC-MS/MS analysis, and thereby, attempts to increase the overall proteome coverage [16]. In total, 13,271 MS/MS spectra were identified, 8,208 (62%) of which were linked to peptides containing methionine. These spectra identified 1,998 unique peptides in 800 proteins (Table 1). In order to reduce the number of possible false positive identification, stringent filtering criteria were used on all peptide-to-spectrum matches (Table 2). These filtering criteria reduced the number of identified and quantified proteins to 531, corresponding to 29% of all ORF products (Table S1). These proteins were assigned to 20 functional categories (Figure 1) based on GO terms [17]. Our protein set covers a wide range of cellular functions, which suggests unbiased sampling of bacterial proteins, a requirement for comparative proteomics studies.

**Table 1:** Synopsis of the protein identification results from the methionine-COFRADIC analysis following stringent filtering of peptide-to-spectrum matches.

Study	Technique	Proteome digestion	Labelling	Obtained spectra	Methionine containing peptides	Unique peptides	Unique proteins	Quantified proteins
**Q212**	Methionine-COFRADIC	Endoproteinase Lys-C	^12^C_3_-propionate	13,271	8,208	1,998	800	531
**Q212Dox**	Methionine-COFRADIC	Endoproteinase Lys-C	^13^C_3_-propionate	13,271	8,208	1,998	800	531

The analysis of the samples, *C. burnetii* cultured in presence of tetracycline (Q212Dox) or absence of the antibiotic (Q212) led to the acquisition of 13,271 MS/MS-spectra. Following filtering, 531 proteins common to both samples were identified.

**Table 2:** The filtering criteria used for protein identification.

Filtering criteria
Withhold peptide-to-spectrum matches with Mascot score≥Mascot's identity score at 99% confidence
Remove peptides shorter than 8 amino acids
Remove peptides with a score less than 10 points above the threshold
Remove peptides with a FALSE ratio

### Differentially regulated bacterial proteins under tetracycline stress

**Figure 2:**
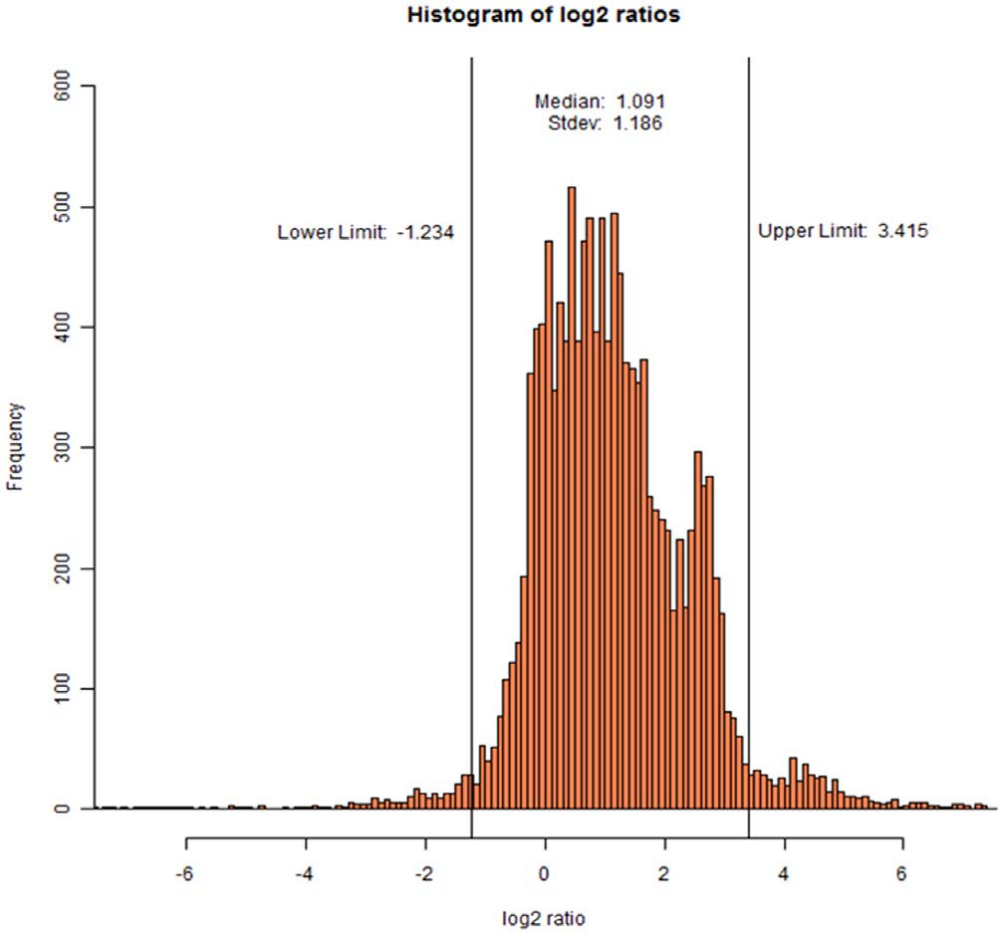
Statistical analysis using 95% confidence settings for determining the limits of protein log2 ratio values linked to differential regulation. These limits are >3.415 and <−1.234. Thus, a protein with a value <−1.234 indicates higher abundance upon antibiotic stress, whereas a protein with a value >3.415 indicates higher abundances in the absence of antibiotic.

The Rover tool developed by the Gevaert lab was used to compare the proteomes of both bacterial samples [18]. Following statistical analysis at 95% confidence, proteins with a protein ratio less than 0.45 or higher than 7.83 were considered as significantly up or down-regulated (p<0.05), with high ratio values pointing to proteins that were more abundant in non-treated bacteria. The broad distribution of protein ratios shown in Figure 2 can be interpreted as though we were dealing with two very different proteomes and thus likely indicates that large proteome adaptations occur under conditions of antibiotic stress.

In total, 24 proteins were found significantly regulated upon doxycycline treatment; 5 proteins were up-regulated, whereas 19 proteins were down-regulated as a result of doxycycline treatment. Table S2 summarises the information obtained through *in silico* analysis of these 24 proteins.

PSORTb v.3.0 was further used to allocate the identified proteins to the cytoplasm, the cytoplasmic membrane, the periplasmic space, the outer membrane or to extracellular sites. 5 proteins were annotated to reside in the cytoplasm, 3 in the cytoplasmic membrane, 1 was in the periplasmic space, but the majority (15) had no known localization. The regulated proteins are involved in energy metabolism (2/24), transport and binding (4/24), protein fate (2/24), amino acid biosynthesis (2/24), biosynthesis of cofactors (1/24), purine and pyrimide (1/24), DNA metabolism (1/24) and detoxification (1/24). Finally, the overrepresentation of proteins with unknown function (10/24) is of particular note (Table S2).

## Discussion

The aim of the present study was to analyze how the proteome profile of *C. burnetii* changed upon tetracycline stress, with the goal of providing insights into as of yet unexplored mechanisms of tetracycline resistance, which might eventually point to protein(s) that could be considered for targeting when developing novel drugs. Comparison of the proteome profiles of the two *C. burnetii* samples indeed pointed to several proteins with regulated expression under tetracycline stress conditions, and these proteins are discussed in the following sections.

### Up-regulated proteins under tetracycline stress conditions

5 proteins were found to be up-regulated upon doxycycline treatment. The predicted cellular functions of these proteins were protein and peptide secretion and trafficking (Q83CL5), energy metabolism, pentose phosphate pathway (Q83DM4), biosynthesis of pantothenate and coenzyme A (Q83EA2), aspartate biosynthesis (Q83A62) and DNA replication (Q83C00).

Signal peptidase I (SPI) (Q83CL5) belongs to a group of serine proteases [19], and plays an essential role in the processing of preproteins carrying a typical N-terminal signal peptide, upon translocation across the cytoplasmic membrane [20]. Previous studies indicated that suppression of the expression of the gene encoding for SPI (lepB) interrupted cell growth and division [21], [22]. Many membrane and secreted bacterial proteins are synthesized as precursors with an N-terminal signal peptide that contains 15 to 30 amino acids. SPases catalyze processing of these signal peptides, thereby allowing protein secretion from the membrane [23], [24]. Bacterial SPases consist of single polypeptides that are anchored in the membrane by one or two transmembrane regions. The most studied SPase is that of *E. coli*, which spans the membrane twice [23], similar to the here identified SPI. The direct link between cell growth and division, and increased expression of SPI, suggests the possible existence of a mechanism of *C. burnetii* to survive under tetracycline stress conditions. Furthermore, the functional relation of this protein with secreted and membrane proteins suggests a possible role for additional activation of such proteins by SPI and thus a more active mode of excretion of the antibiotic. Type I SPases can be irreversibly inhibited by certain penem compounds that acylate the serine residue in the active site. Since SPase activity was found to be essential for cell viability, SPases are considered to be potential targets for developing novel antibacterial agents [19], [25]. Our study also places *C. burnetii* SPI as a possible target for inhibition by penem derivatives.

Transaldolase (Q83DM4) functions in the pentose phosphate pathway and is almost ubiquitously found in the three domains of life (Archaea, Bacteria, and Eukarya). It provides cells with important redox cofactors such as NADPH, building blocks for nucleotide and nucleic acid biosynthesis [26]. Although increased expression rates of this protein in novobiocyn-resistant *Staphylococcus aureus* strains has been reported, its increase was not associated with a possible mechanism of bacterial resistance against antibiotics [27]. It is however likely though that transaldolase over-expression upon doxycycline administration can balance the probably increased demand of the bacterium for energy to excrete the antibiotic.

The DNA polymerase III alpha subunit (Q83C00) might also be associated with a molecular mechanism of antibiotic resistance. This protein is considered to be the main mediator of pathogen survival through induction of mutagenicity. It contributes to the creation of mutants that are able to address the host immune system and administered antibiotics [28].

3-methyl-2-oxobutanoate hydroxymethyltransferase (Q83EA2) participates in the biosynthesis of pantothenate and coenzyme A (see http://cmr.jcvi.org/cgi-bin/CMR/CmrHomePage.cgi). Coenzyme A has been implicated in resistance mechanisms against aminoglycoside-based antibiotics (AGs). AGs bind to the 30S subunit of the prokaryotic ribosome and interrupt bacterial protein synthesis, a mode-of-action that is similar to that of tetracycline. Coenzyme A is directly involved in the enzymatic covalent modification of AGs, which causes a strong decrease in the AG's binding affinity to the prokaryotic ribosome [29].The fact that the 3-methyl-2-oxobutanoate hydroxymethyltransferase is over-expressed upon doxycycline treatment possibly indicates a similar function of *C. burnetii's* coenzyme A to tetracycline resistance.

Methyltetrahydropteroyltriglutamate–homocysteine methyltransferase (Q83A62) was also over-expressed upon doxycycline treatment. This enzyme is involved in methionine biosynthesis (http://cmr.jcvi.org/cgi-bin/CMR/CmrHomePage.cgi). Despite extensive literature search however, no connection between methionine biosynthesis and antibiotic resistance mechanisms was found.

### Down-regulated proteins under tetracycline stress conditions

19 proteins were down-regulated under tetracycline stress conditions. Their predicted cellular functions were diverse, including protein folding and stabilization-heat response (Q83CE9), aspartate biosynthesis (P24703), detoxification (Q83AQ8), threonine catabolic process (Q83F39), purine ribonucleoside salvage (Q83FC4), transport and binding (Q83BF9, Q82OW5, Q83DI7 and Q83AW2) and unknown function (B5QSC3, B5QSC0, Q83A96, Q83EA1, Q83EJ9, Q83A79, Q83D04, Q83DR4, Q83B41 and Q83CL9). One example of the latter is ScvA (B5QSC0), a highly basic DNA binding protein specific for the small cell variant (SCV) of *C. burnetii* and believed to play role in chromatin condensation [30]. Interestingly, ScvA was found down-regulated in a similar study but using levofloxacine stress [31], and its down-regulation under tetracycline stress conditions agrees with the levofloxacine study in which it was hypothesized that SCVs of *C. burnetii* are predominantly present in antibiotic-free cultures. The putative uncharacterized protein B5QSC3 was also down-regulated upon antibiotic stress. In fact, 8 out of the 19 down-regulated proteins have currently no known function, which highlights the need for future studies towards characterizing *C. burnetii*' s proteome.

Down-regulation of the phosphoenolpyruvate-protein phosphotransferase (Q83BF9) is of particular interest. This protein was identified in a previous study in which a persistent model of *C. burnetii* infection was compared with an acute one, and found to be only present in the acute strain [32].

Levels of the ferrous iron transport protein B (Q83AW2) were also found to be down-regulated. Iron (Fe^2+^) is a cofactor in a variety of biological processes and many bacteria transport iron through Feo systems (ferrous iron transport). It has been suggested that bacterial iron metabolism is relevant for the activity of antibiotics and that mutations in proteins important for this metabolism might challenge bacterial antibiotic susceptibility [33]. In this respect, it was shown that deletion of the gene encoding for ferric reductase confers resistance to antibiotics in *Pseudomonas*, whereas its over-expression accelerates antibiotics-induced cell death [34]. Many pathogens show reduced pathogenicity in the absence of this protein, reflecting the importance of iron absorption [35]. It has been suggested that elevated iron transport triggers the activation of immune cytokines [36], [37]. Taken together, our and previous data hint to the possibility that tetracycline-challenged *C. burnetii* down-regulate iron transport to avoid cytokine activation.

Although the regulated proteins here identified could not be directly linked to known mechanisms of antibiotic resistance, closer inspection revealed several known proteins that lie on the limits of statistically significant under/over-expression. A characteristic protein is the ribosome associated factor Y (pY, Q83DI6) found down-regulated upon doxycycline treatment (ratio value of 7.54). Ribosomes are clearly of utmost importance for controlling protein expression. The pY protein has been studied in *E. coli* and is expressed under low temperature [38] or excessive cell concentration [39]. It inhibits translation, probably analogous to the action of tetracycline, thus by preventing the association of aminoacyl-tRNA in the ribosomal A site [40]. By lowering pY expression, *C. burnetii* might try to increase protein synthesis, thereby addressing protein synthesis inhibition by tetracycline. In support of this hypothesis are the results of the study by Agafonov and co-workers who demonstrated that association of tetracycline with the ribosomal A site in *E. coli* was not affected by the presence of large amounts of pY [40]. Also the reverse hold true since the interaction of pY with ribosomes was not affected by tetracycline [41]. Therefore, protein inhibiting protein synthesis and antibiotics do not interfere with each other for association with ribosomes, thus excluding possible competition for the ribosomal A site.

Of further note is that the vast majority of the proteins with predicted role in translation and protein biosynthesis (69 out of 77 proteins identified) had protein ratio values that place them at the border of statistically significant over-expressed proteins upon doxycycline treatment. Furthermore, stretching the limits for statistical significance a bit, of the additional 158 proteins with ratio values up to 1.1, the predicted cellular function of 52 of them was translation and protein biosynthesis. This observation, coupled to the mode of action of tetracyclines leads us to suspect the existence of a mechanism protective for bacterial ribosomes, a well-known tetracycline resistance mechanism. Tetracycline resistance by expressing ribosomal protection proteins (RPPs) is achieved by weakening the interaction of tetracycline and the ribosome with subsequent antibiotic release [42]. RPPs free ribosomes from the inhibitory effects of drugs, such that aa-tRNA can re-bind to the A-sites and protein synthesis can continue. Although it is believed that RPPs work as tetracycline resistant elongation factors to carry out protein synthesis in the presence of tetracycline, substitution experiments have ruled out this hypothesis [43]. Common characteristics of RPPs are their molecular size (approximately 72.5 kDa) and their high amino acid sequence homology to translation elongation factors EF-Tu and EF-G GTPases [44]. In this context, the here identified elongation factor 4 (Q83BK3) is predicted to be required for accurate and efficient protein synthesis under certain stress conditions. This protein possesses all the main characteristics of a RPP, it is classified as a GTPase, its molecular weight is 67.8 kDa and it shares high sequence homology to elongation factor proteins Tu and G. In addition, elongation factor 4 was shown to contribute to tellurite resistance in *Escherichia coli* without an apparent role in the fidelity of protein synthesis, suggesting a role in tetracycline resistance [45].

As mentioned before, active efflux of tetracycline from bacteria is the second most predominant tetracycline resistance mechanism in clinical settings. This efflux mainly occurs via integral membrane transporters that belong to the major facilitator transporter superfamily (MFS) [46]. These efflux pumps span the lipid bilayer of the inner cell membrane 12 to 14 times. Based on homology to known transporters, the membrane spanning regions of these proteins are predicted to be helical, forming a water-filled channel surrounded by six transmembrane helices. Tetracyclines are predicted to pass through this channel and exchanged for H^+^ [47]. One MFS protein (Q83EP3) and a Na^+^-driven multidrug efflux pump (Q83DC6) have been identified in our study. *In silico* analysis of these proteins identified 11 and 12 transmembrane helices, respectively (http://www.cbs.dtu.dk/services/TMHMM-2.0/), and thus, these proteins could be capable of secreting tetracycline.

The fact that certain antibiotics may be signaling molecules at low concentrations [48], [49], [50], [51], [52], along with the idea that the original function of antibiotic resistance determinants is not counteracting the activity of drugs [53], [54], leads to the suggestion that antibiotic resistance elements might have been primarily selected for playing roles relevant for microbial physiology, while their activity in avoiding the action of antimicrobials is secondary to these roles [53], [55], [56], [57]. In support of this comes the finding that building up the intrinsic antibiotic resistance phenotype requires the concerted activity of a large number of elements, several of which play primary roles in microbial physiology, including proteins and elements of the bacterial metabolic networks such as components of the electron transport chain or of the metabolism of amino acids, fatty acids or nucleotides [51], [58], [59], [60]. The findings from our study further support this concept. Even though there was no statistically significant regulation of proteins known to participate in antibiotic resistance mechanisms, several proteins of the pathogen's metabolism were differentially regulated, indicating a concerted activity in the bacterium to modulate antibiotic resistance using its metabolism. An analogous conclusion can be drawn from a previous proteomics study on a levofloxacine-resistant *C. burnetii* strain [31]. Taking into account that cellular processes are carried out by proteins and not DNA, we propose that, at least concerning *C. burnetii*, when referring to antibiotic resistance, one may not simply limit the potential mechanisms of resistance to those already known from other studies. Instead, we must consider that resistance to an antibiotic is not exclusively the result of a mutated gene, but rather a multifaceted process as indicated by the proteome rearrangements observed in this study.

### Conclusions

Quantitative proteome comparison of a *C. burnetii* strain propagated under tetracycline stress conditions with an antibiotic-free cultured strain identified 5 up-regulated and 19 down-regulated proteins upon tetracycline stress. The predicted cellular functions of the differentially regulated proteins include secretion and trafficking, energy metabolism, amino acid biosynthesis, DNA metabolism and protein fate. Several plausible hypotheses have been formed to establish a link between these proteins and tetracycline stress conditions. Our further analysis of the identified proteins indicated the proteome potential of *C. burnetii* to resist tetracycline. Higher antibiotic concentrations or a proteome analysis of a tetracycline resistant *C. burnetii* clinical isolate could further indicate proteins that are directly implicated in tetracycline resistance mechanisms, leading to more effective drug treatment and minimization of treatment failures.

## Materials and Methods

### *C. burnetii* culture in the presence of tetracycline

*C. burnetii* Q212 (CbuG_Q212) phase II Q fever reference strain was propagated in confluent African Green Monkey kidney fibroblasts (Vero; ATCC no. CCL- 81) in 225 cm^2^ angled neck flasks (Corning Inc., U.S.A.). Infected cells were cultured in minimum essential medium (MEM; Gibco Laboratories) supplemented with 4% fetal bovine serum (FBS; Gibco Laboratories and 2 mM L-glutamine (Gibco Laboratories) at 35°C in a 5% CO_2_ incubator. Infection was monitored by Gimenez staining [61]. Briefly, Vero cells were inoculated and cultured in medium free of antibiotics until 90% of the cells were infected. The supernatant was removed and infected cells were harvested and centrifuged at 1000×*g* for 15 min. The pellet was re-suspended in fresh culture medium, and the cells were disrupted by three freeze-thaw cycles (from −210°C to 37°C). After centrifugation at 1000×*g* for 15 min, the supernatant with the liberated bacteria from the infected cells was used to infect fresh Vero cell monolayers. The inoculum was removed after 1 h of incubation at 35°C in a 5% CO_2_ atmosphere, the cells were washed with culture medium in order to eliminate unphagocytosed bacteria, and fresh medium containing 0.2 mg/L of doxycycline (DOX) was added. The monolayer was incubated at 35°C in a 5% CO_2_ atmosphere. The supernatant was removed every three days, and fresh medium containing 0.2 mg/L of DOX was added. The same procedure was followed with constantly increasing concentrations of the antibiotic until bacteria were collected for inoculation titration test and MIC determination [62]. Parallel cultures of the same strain were kept at all times undergoing the same experimental procedures with absence of the antibiotic so as to serve as the reference strain (Q212).

The antibiotics used in this study was doxycycline hydrochloride (DOX) (α-6-deoxy-5-hydroxytetracycline, Vibramycin, Hyclate, Calbiochem, Merck KGaA, Germany). DOX was directly diluted into freshly prepared culture medium to the appropriate concentration. Culture media containing the antibiotics were sterilized by filtration (pore size: 0.22 µm) and stored at 4°C. DOX was shown, by shell vial assay (data not included), to be nontoxic to Vero cells to concentrations up to 128 mg/L.

### *C. burnetii* inoculation titration test and MIC determination

To determine the effect of tetracycline in the culture medium on the MIC of *C. Burnetii*, inoculation titration tests and Minimum Inhibitory Concentration determinations were performed as described previously [31]. After six days of incubation at 35°C, 5% CO_2_, the MIC of DOX was determined by direct immunofluorescence as the concentration of the antibiotic at which there were no intracellular bacteria observed following a six days incubation period. Even though during MIC determination tests bacteria were initially challenged by an initial concentration well beyond the MIC mentioned in the literature for *C. burnetii* Q212 (2 mg/L; initial antibiotic concentration, 0.125 mg/L), negative controls (no antibiotic present) were included at all times. During *C. burnetii* culture under tetracycline stress conditions (15 weeks), the observed MIC for doxycycline increased from 2 mg/l to 16 mg/l (MIC Q212Dox: 16 mg/l), while bacteria propagated in the absence of antibiotic for the same time period, did not show a MIC change (MIC Q212: 2 mg/l).

### Bacteria and protein isolation

Bacteria propagated with DOX (Q212Dox) and without DOX (Q212) were mass cultured in parallel and under identical conditions for 10 days (with the exception of doxycycline present in Q212Dox) and finally isolated from their host cells using renographin (Ultravist 370; 0.769 g/mL iopromide; Schering) density gradient ultracentrifugation as described [17]. Renographin clean bacteria re-suspended in K36 buffer (16.5 mM KH_2_PO_4_, 33.5 mM K_2_HPO_4_, 100 mM KCl, 15.5 mM NaCl, pH 7.0) containing a protease inhibitor cocktail (Sigma-Aldrich) underwent five 5-min freeze-thaw cycles (from −210°C to 37°C) and the protein concentration was determined by the Bradford assay. All manipulations involving viable bacteria were performed in a Biosafety Level III laboratory. It has to be noted that upon completion of the experiments all bacteria cultured under tetracycline stress conditions were killed by incubation in 8% formaldehyde at room temperature for 24 h followed by autoclaving. Prior to disposal, bacteria were used to inoculate Vero cells to check their inactivation.

### Isolation and identification of methionine-containing peptides by COFRADIC

The methionine-COFRADIC procedure was performed on both Q212Dox and Q212 whole lysates as described [16]. In brief, proteomes were digested with endoproteinase Lys-C and the peptides were labeled by N-propionylation. The Q212 proteome digest was labeled with ^12^C_3_-propionate whereas the Q212Dox proteome digest with ^13^C_3_-propionate. Each peptide thus had a label on its N-terminal alpha-amino group and on its C-terminal lysine epsilon-amino group, evoking a difference of 6 Da between light and heavy peptides. Samples were mixed and analyzed by LC-MS/MS on an Ultimate 3000 HPLC system (Dionex, Amsterdam, The Netherlands) in-line connected to a LTQ Orbitrap XL mass spectrometer (Thermo Electron, Bremen, Germany). Instrument settings for LC-MS/MS analysis and generation of MS/MS peak lists were as described [63]. MS/MS peak lists were searched with Mascot using the Mascot Daemon interface (version 2.2.0, Matrix Science). The Mascot search parameters were as follows. Searches were performed in a *Coxiella burnetii* database downloaded from Uniprot on June 17^th^, 2009 (containing 1,815 protein entries). Lys-C/P was set as the used protease with one missed cleavage allowed, and the mass tolerance on the precursor ion was set to ±10 ppm and on fragment ions to ±0.5 Da. *S*-carbamidomethylation of cysteine and oxidation of methionine (to its sulfoxide) were set as fixed modifications. In addition, Mascot's C13 setting was set to 1. The light and heavy labels were defined in Mascot Distiller's quantitation method. Peptide quantifications were carried out using the Mascot Distiller Quantitation Toolbox (version 2.2.1). The quantification method details were as follows: constrain search: yes, protein ratio type: average, report detail: yes, minimum peptides: 1, protocol: precursor, allow mass time match: yes, allow elution shift: no, all charge states: yes. Ratios for identified proteins were calculated by comparing the XIC peak areas of all matched light peptides with those of the heavy peptides, and the results were verified by visual inspection of MS spectra with the in-house developed Rover tool [18]. All identified MS/MS spectra are publicly available in the PRIDE database (http:www.ebi.ac.uk/pride) under the experiment number 18640 (note that currently only referees can examine these data using the login: review23777, and password: x_MgR4R$).

### Protein analysis by bioinformatics tools

Analysis of the amino acid sequences of all identified proteins was carried out using several Web-based software tools, freely accessible from the ExPASy Proteomics Server of the Swiss Institute of Bioinformatics (SIB) (http://au.expasy.org/). The purpose of this analysis was to calculate the molecular weight (MW) and isoelectric point (p*I*) of each protein using the software ProtParam Tool (http://au.expasy.org/tools/protparam.html) and Compute pI/M_w_ Tool (http://au.expasy.org/tools/pi_tool.html).

## Supporting Information

Table S1 List of proteins identified in both *C. burnetii* samples. All 531 proteins were identified in both samples (i. e. *C. burnetii* cultured in presence of tetracycline and *C. burnetii* cultured with no antibiotic present) in different expression levels. Proteins are categorized according to their predicted cellular function; their ratio value, gene locus, molecular weight (MW), isoelectric point and predicted cellular function are indicated.
(DOCX)

Table S2 Proteins that were significantly over-expressed in *C. burnetii* when cultured in presence of tetracycline (Q212Dox) or in *C. burnetii* when cultured with no antibiotic (Q212).
(DOCX)

## References

[1] Omsland A, Beare PA, Hill J, Cockrell DC, Howe D (2011). Isolation from Animal Tissue and Genetic Transformation of Coxiella burnetii Are Facilitated by an Improved Axenic Growth Medium.. Appl Environ Microbiol.

[2] Omsland A, Cockrell DC, Howe D, Fischer ER, Virtaneva K (2009). Host cell-free growth of the Q fever bacterium Coxiella burnetii.. Proc Natl Acad Sci U S A.

[3] Derrick EH (1937). Q” fever, a new fever entity: clinical features, diagnosis and laboratory investigation.. Medical Journal of Australia.

[4] Tissot-Dupont H, Vaillant V, Rey S, Raoult D (2007). Role of sex, age, previous valve lesion, and pregnancy in the clinical expression and outcome of Q fever after a large outbreak.. Clin Infect Dis.

[5] Gami AS, Antonios VS, Thompson RL, Chaliki HP, Ammash NM (2004). Q fever endocarditis in the United States.. Mayo Clin Proc.

[6] Angelakis E, Raoult D (2010). Q Fever.. Vet Microbiol.

[7] Joshi N, Miller DQ (1997). Doxycycline revisited.. Arch Intern Med.

[8] Maurin M, Raoult D (1999). Q fever.. Clin Microbiol Rev.

[9] Rolain JM, Mallet MN, Raoult D (2003). Correlation between serum doxycycline concentrations and serologic evolution in patients with Coxiella burnetii endocarditis.. J Infect Dis.

[10] Rolain JM, Lambert F, Raoult D (2005). Activity of telithromycin against thirteen new isolates of C. burnetii including three resistant to doxycycline.. Ann N Y Acad Sci.

[11] Turck WP, Howitt G, Turnberg LA, Fox H, Longson M (1976). Chronic Q fever.. Q J Med.

[12] Chopra I, Hawkey PM, Hinton M (1992). Tetracyclines, molecular and clinical aspects.. J Antimicrob Chemother.

[13] Brodersen DE, Clemons WM, Carter AP, Morgan-Warren RJ, Wimberly BT (2000). The structural basis for the action of the antibiotics tetracycline, pactamycin, and hygromycin B on the 30S ribosomal subunit.. Cell.

[14] Thaker M, Spanogiannopoulos P, Wright GD (2010). The tetracycline resistome.. Cell Mol Life Sci.

[15] Beare PA, Unsworth N, Andoh M, Voth DE, Omsland A (2009). Comparative genomics reveal extensive transposon-mediated genomic plasticity and diversity among potential effector proteins within the genus Coxiella.. Infect Immun.

[16] Gevaert K, Van Damme J, Goethals M, Thomas GR, Hoorelbeke B (2002). Chromatographic isolation of methionine-containing peptides for gel-free proteome analysis: identification of more than 800 Escherichia coli proteins.. Mol Cell Proteomics.

[17] Samoilis G, Psaroulaki A, Vougas K, Tselentis Y, Tsiotis G (2007). Analysis of whole cell lysate from the intercellular bacterium Coxiella burnetii using two gel-based protein separation techniques.. J Proteome Res.

[18] Colaert N, Helsens K, Impens F, Vandekerckhove J, Gevaert K (2010). Rover: a tool to visualize and validate quantitative proteomics data from different sources.. Proteomics.

[19] Paetzel M, Dalbey RE, Strynadka NC (1998). Crystal structure of a bacterial signal peptidase in complex with a beta-lactam inhibitor.. Nature.

[20] Tuteja R (2005). Type I signal peptidase: an overview.. Arch Biochem Biophys.

[21] Dalbey RE, Wickner W (1985). Leader peptidase catalyzes the release of exported proteins from the outer surface of the Escherichia coli plasma membrane.. J Biol Chem.

[22] Date T (1983). Demonstration by a novel genetic technique that leader peptidase is an essential enzyme of Escherichia coli.. J Bacteriol.

[23] Dalbey RE, Lively MO, Bron S, van Dijl JM (1997). The chemistry and enzymology of the type I signal peptidases.. Protein Sci.

[24] Date T, Wickner W (1981). Isolation of the Escherichia coli leader peptidase gene and effects of leader peptidase overproduction in vivo.. Proc Natl Acad Sci U S A.

[25] Black MT, Bruton G (1998). Inhibitors of bacterial signal peptidases.. Curr Pharm Des.

[26] Samland AK, Sprenger GA (2009). Transaldolase: from biochemistry to human disease.. Int J Biochem Cell Biol.

[27] Vinnikov AI, Babenko Iu S (1989). [Features of glycolysis and pentose phosphate pathway in novobiocin sensitive and novobiocin resistant staphylococci].. Antibiot Khimioter.

[28] Boshoff HI, Reed MB, Barry CE, Mizrahi V (2003). DnaE2 polymerase contributes to in vivo survival and the emergence of drug resistance in Mycobacterium tuberculosis.. Cell.

[29] Hu X, Norris AL, Baudry J, Serpersu EH (2011). Coenzyme A Binding to the Aminoglycoside Acetyltransferase (3)-IIIb Increases Conformational Sampling of Antibiotic Binding Site.. Biochemistry.

[30] Heinzen RA, Howe D, Mallavia LP, Rockey DD, Hackstadt T (1996). Developmentally regulated synthesis of an unusually small, basic peptide by Coxiella burnetii.. Mol Microbiol.

[31] Vranakis I, De Bock PJ, Papadioti A, Tselentis Y, Gevaert K (2011). Identification of potentially involved proteins in levofloxacin resistance mechanisms in Coxiella burnetii.. J Proteome Res.

[32] Vranakis I, De Bock PJ, Papadioti A, Samoilis G, Tselentis Y (2011). Unraveling Persistent Host Cell Infection with Coxiella burnetii by Quantitative Proteomics.. J Proteome Res.

[33] Kohanski MA, Dwyer DJ, Hayete B, Lawrence CA, Collins JJ (2007). A common mechanism of cellular death induced by bactericidal antibiotics.. Cell.

[34] Yeom J, Imlay JA, Park W (2010). Iron homeostasis affects antibiotic-mediated cell death in Pseudomonas species.. J Biol Chem.

[35] Stojiljkovic I, Cobeljic M, Hantke K (1993). Escherichia coli K-12 ferrous iron uptake mutants are impaired in their ability to colonize the mouse intestine.. FEMS Microbiol Lett.

[36] Chen L, Xiong S, She H, Lin SW, Wang J (2007). Iron causes interactions of TAK1, p21ras, and phosphatidylinositol 3-kinase in caveolae to activate IkappaB kinase in hepatic macrophages.. J Biol Chem.

[37] Xiong S, She H, Takeuchi H, Han B, Engelhardt JF (2003). Signaling role of intracellular iron in NF-kappaB activation.. J Biol Chem.

[38] Agafonov DE, Kolb VA, Spirin AS (2001). A novel stress-response protein that binds at the ribosomal subunit interface and arrests translation.. Cold Spring Harb Symp Quant Biol.

[39] Maki Y, Yoshida H, Wada A (2000). Two proteins, YfiA and YhbH, associated with resting ribosomes in stationary phase Escherichia coli.. Genes Cells.

[40] Agafonov DE, Kolb VA, Spirin AS (2001). Ribosome-associated protein that inhibits translation at the aminoacyl-tRNA binding stage.. EMBO Rep.

[41] Agafonov DE, Kolb VA, Nazimov IV, Spirin AS (1999). A protein residing at the subunit interface of the bacterial ribosome.. Proc Natl Acad Sci U S A.

[42] Connell SR, Tracz DM, Nierhaus KH, Taylor DE (2003). Ribosomal protection proteins and their mechanism of tetracycline resistance.. Antimicrob Agents Chemother.

[43] Burdett V (1996). Tet(M)-promoted release of tetracycline from ribosomes is GTP dependent.. J Bacteriol.

[44] Taylor DE, Chau A (1996). Tetracycline resistance mediated by ribosomal protection.. Antimicrob Agents Chemother.

[45] Shoji S, Janssen BD, Hayes CS, Fredrick K (2010). Translation factor LepA contributes to tellurite resistance in Escherichia coli but plays no apparent role in the fidelity of protein synthesis.. Biochimie.

[46] Paulsen IT, Brown MH, Skurray RA (1996). Proton-dependent multidrug efflux systems.. Microbiol Rev.

[47] Tamura N, Konishi S, Yamaguchi A (2003). Mechanisms of drug/H+ antiport: complete cysteine-scanning mutagenesis and the protein engineering approach.. Curr Opin Chem Biol.

[48] Linares JF, Gustafsson I, Baquero F, Martinez JL (2006). Antibiotics as intermicrobial signaling agents instead of weapons.. Proc Natl Acad Sci U S A.

[49] Yim G, Wang HH, Davies J (2006). The truth about antibiotics.. Int J Med Microbiol.

[50] Yim G, Wang HH, Davies J (2007). Antibiotics as signalling molecules.. Philos Trans R Soc Lond B Biol Sci.

[51] Fajardo A, Martinez JL (2008). Antibiotics as signals that trigger specific bacterial responses.. Curr Opin Microbiol.

[52] Yergeau E, Lawrence JR, Waiser MJ, Korber DR, Greer CW (2010). Metatranscriptomic analysis of the response of river biofilms to pharmaceutical products, using anonymous DNA microarrays.. Appl Environ Microbiol.

[53] Martinez JL, Fajardo A, Garmendia L, Hernandez A, Linares JF (2009). A global view of antibiotic resistance.. FEMS Microbiol Rev.

[54] Martinez JL, Sanchez MB, Martinez-Solano L, Hernandez A, Garmendia L (2009). Functional role of bacterial multidrug efflux pumps in microbial natural ecosystems.. FEMS Microbiol Rev.

[55] Martinez JL (2008). Antibiotics and antibiotic resistance genes in natural environments.. Science.

[56] Aminov RI (2009). The role of antibiotics and antibiotic resistance in nature.. Environ Microbiol.

[57] Fajardo A, Linares JF, Martinez JL (2009). Towards an ecological approach to antibiotics and antibiotic resistance genes.. Clin Microbiol Infect.

[58] Breidenstein EB, Khaira BK, Wiegand I, Overhage J, Hancock RE (2008). Complex ciprofloxacin resistome revealed by screening a Pseudomonas aeruginosa mutant library for altered susceptibility.. Antimicrob Agents Chemother.

[59] Tamae C, Liu A, Kim K, Sitz D, Hong J (2008). Determination of antibiotic hypersensitivity among 4,000 single-gene-knockout mutants of Escherichia coli.. J Bacteriol.

[60] Dotsch A, Becker T, Pommerenke C, Magnowska Z, Jansch L (2009). Genomewide identification of genetic determinants of antimicrobial drug resistance in Pseudomonas aeruginosa.. Antimicrob Agents Chemother.

[61] Gimenez DF (1964). Staining Rickettsiae in Yolk-Sac Cultures.. Stain Technol.

[62] Maurin M, Raoult D (1997). Bacteriostatic and bactericidal activity of levofloxacin against Rickettsia rickettsii, Rickettsia conorii, ‘Israeli spotted fever group rickettsia’ and Coxiella burnetii.. J Antimicrob Chemother.

[63] Ghesquiere B, Colaert N, Helsens K, Dejager L, Vanhaute C (2009). In vitro and in vivo protein-bound tyrosine nitration characterized by diagonal chromatography.. Mol Cell Proteomics.

