# Supplementary material for: Quantitative Proteome Profiling of C. burnetii under Tetracycline Stress Conditions
Source: PLoS One. 2012 Mar 16;7(3):e33599. doi: 10.1371/journal.pone.0033599 (PMC3306420; doi:10.1371/journal.pone.0033599)
Supplement: Table S1 — List of proteins identified in both C. burnetii samples. All 531 proteins were identified in both samples (i. e. C. burnetii cultured in presence of tetracycline and C. burnetii cultured with no antibiotic present) in different expression levels. Proteins are categorized according to their predicted cellular function; their ratio value, gene locus, molecular weight (MW), isoelectric point and predicted cellular function are indicated. (DOCX) [file pone.0033599.s001.docx]

| **Table S1.** List of proteins identified in both *C. burnetii* samples. | | | | | | |
| --- | --- | --- | --- | --- | --- | --- |
| **Accession No** | **Uniprot Description** | **Light/Heavy protein ratio** | **Gene locus** | **M/W** | **pI** | **Predicted Cellular Function** |
| Q83CL9 | Hypothetical exported protein | 47.6504 | CBU_1095 | 17.9 | 7.4 | Unknown |
| Q83B41 | Hypothetical cytosolic protein | 25.5243 | CBU_1677 | 17.3 | 6.6 | Unknown |
| Q83DR4 | Putative uncharacterized protein | 21.2329 | CBU_0632 | 11.8 | 4.4 | Unknown |
| Q83D04 | Rhodanese-related sulfurtransferase | 21.1506 | CBU_0943 | 13.9 | 8.2 | Unknown |
| Q83A79 | Hypothetical exported protein | 20.5577 | CBU_2029 | 24.4 | 10.1 | Unknown |
| Q83EJ9 | IcmH | 12.1414 | CBU_0321 | 29.1 | 6 | Unknown |
| Q83EA1 | Putative uncharacterized protein | 10.4498 | CBU_0425 | 51.8 | 5.7 | Unknown |
| Q83A96 | Hypothetical exported protein | 10.2468 | CBU_2010 | 12 | 10.6 | Unknown |
| B5QSC0 | ScvA | 9.3417 | CBU_1267.1 | 3.6 | 12.1 | Unknown |
| B5QSC3 | Putative uncharacterized protein | 9.0779 | CBU_1280.1 | 9.8 | 8.8 | Unknown |
| Q83B63 | IcmX | 6.7314 | CBU_1652 | 41.3 | 6.3 | Unknown |
| Q83D25 | Hypothetical cytosolic protein | 6.6968 | CBU_0921 | 11.7 | 6.9 | Unknown |
| Q83CS6 | ATPase | 6.3323 | CBU_1027 | 50.7 | 10 | Unknown |
| Q83CF8 | Tetratricopeptide repeat family protein | 6.2479 | CBU_1160 | 45.5 | 6.5 | Unknown |
| Q83F37 | UPF0234 protein CBU_0114 | 5.756 | CBU_0114 | 18.1 | 8.9 | Unknown |
| Q83F03 | UPF0289 protein CBU_0150 | 5.5129 | CBU_0150 | 29.7 | 8.1 | Unknown |
| Q83AA2 | Putative ribosome maturation factor rimN | 5.4718 | CBU_2003 | 20.5 | 5.8 | Unknown |
| Q83F57 | Tol system periplasmic component | 5.3709 | CBU_0092 | 32 | 5.4 | Unknown |
| Q83C94 | Hypothetical ATPase | 5.2016 | CBU_1234 | 47.5 | 8.1 | Unknown |
| Q83E32 | DNase, TatD family | 5.0332 | CBU_0502 | 25.3 | 6.6 | Unknown |
| Q83E62 | Putative uncharacterized protein | 4.8539 | CBU_0469 | 7.3 | 4.5 | Unknown |
| Q83AC0 | Hypothetical exported protein | 4.6215 | CBU_1984 | 13.7 | 6.9 | Unknown |
| Q83CY8 | Putative peroxiredoxin bcp | 4.4483 | CBU_0963 | 16.8 | 8 | Unknown |
| Q83C39 | Hypothetical membrane associated protein | 4.3815 | CBU_1295 | 12.5 | 9.7 | Unknown |
| Q93N45 | Putative uncharacterized protein | 4.3478 | CBU_0698 | 46.2 | 9.9 | Unknown |
| Q83FB9 | Putative uncharacterized protein | 4.1497 | CBU_0021 | 9.3 | 8.4 | Unknown |
| Q83E42 | DNA Polymerase X family | 4.1415 | CBU_0490 | 65.7 | 10.1 | Unknown |
| Q83B15 | Putative uncharacterized protein | 4.0326 | CBU_1705 | 12.7 | 10.3 | Unknown |
| Q83B28 | Transcriptional regulator | 3.9855 | CBU_1691 | 12.2 | 10.8 | Unknown |
| Q83F35 | S-adenosyl-L-methionine-dependent methyltransferase mraW | 3.9424 | CBU_0116 | 34.9 | 10 | Unknown |
| Q83F36 | Protein mraZ | 3.9085 | CBU_0115 | 17.3 | 5 | Unknown |
| Q83EN6 | Amidinotransferase family protein | 3.5098 | CBU_0279 | 31.8 | 6.3 | Unknown |
| Q83CI7 | IscU | 3.3542 | CBU_1128 | 13.7 | 5.9 | Unknown |
| Q83EN9 | Rubredoxin-NAD(+) reductase | 3.2709 | CBU_0276 | 41.9 | 8.3 | Unknown |
| Q83E24 | Putative uncharacterized protein | 3.234 | CBU_0510 | 11.2 | 5.2 | Unknown |
| Q83AZ1 | Hypothetical exported protein | 3.1453 | CBU_1735 | 40.5 | 6.5 | Unknown |
| Q83AL8 | Hypothetical membrane associated protein | 3.1266 | CBU_1865 | 20.2 | 9.7 | Unknown |
| Q83A53 | Hypothetical cytosolic protein | 3.1145 | CBU_2057 | 38.4 | 6.4 | Unknown |
| Q83CQ3 | Putative competence-damage protein | 3.1129 | CBU_1055 | 17.6 | 9.9 | Unknown |
| Q83AL4 | Hypothetical exported protein | 3.0896 | CBU_1869 | 24.7 | 9.4 | Unknown |
| Q83AX6 | Putative uncharacterized protein | 3.076 | CBU_1751 | 48.8 | 8.1 | Unknown |
| Q83AL3 | 2-nonaprenyl-3-methyl-6-methoxy-1,4-benzoquinol hydroxylase | 3.0621 | CBU_1870 | 23.9 | 7.2 | Unknown |
| Q83DQ8 | Dihydrolipoamide acetyltransferase component of pyruvate dehydrogenase complex | 3.0578 | CBU_0638 | 40.8 | 4.9 | Unknown |
| Q83CA2 | NAD-specific glutamate dehydrogenase | 3.055 | CBU_1226 | 185.9 | 7 | Unknown |
| Q83E96 | Aminomethyltransferase family protein | 3.0075 | CBU_0431 | 28.8 | 7.3 | Unknown |
| Q83EU4 | Peptidase, C40 family | 2.9954 | CBU_0215 | 58 | 6.5 | Unknown |
| Q83AX3 | Uncharacterized protein CBU_1754 | 2.9738 | CBU_1754 | 22.3 | 4.7 | Unknown |
| P53591 | Succinyl-CoA ligase [ADP-forming] subunit alpha | 2.9651 | CBU_1396 | 30.6 | 5.3 | Unknown |
| Q83DY0 | Hypothetical cytosolic protein | 2.8503 | CBU_0560 | 46.4 | 7 | Unknown |
| Q83DP1 | UPF0133 protein CBU_0656 | 2.8349 | CBU_0656 | 12.1 | 4.6 | Unknown |
| Q83BX1 | Hypothetical exported protein | 2.7571 | CBU_1366 | 12.3 | 9.2 | Unknown |
| Q83DJ5 | Uridine phosphorylase | 2.6845 | CBU_0735 | 31.1 | 7.4 | Unknown |
| Q83AU1 | Hypothetical cytosolic protein | 2.6758 | CBU_1788 | 22.3 | 10.2 | Unknown |
| Q83A85 | Putative uncharacterized protein | 2.616 | CBU_2023 | 9.3 | 9.1 | Unknown |
| Q83F83 | Rhodanese-related sulfurtransferase | 2.5888 | CBU_0065 | 14.1 | 5.2 | Unknown |
| Q83BN8 | Putative uncharacterized protein | 2.5109 | CBU_1465 | 18 | 9.7 | Unknown |
| Q83DE2 | Adenosine 5'-monophosphoramidase | 2.5106 | CBU_0796 | 12.4 | 6.7 | Unknown |
| Q83DN9 | Uncharacterized protein CBU_0658 | 2.4517 | CBU_0658 | 15.9 | 4.5 | Unknown |
| Q83BH1 | Non-proteolytic protein, peptidase family M23 | 2.4329 | CBU_1537 | 42 | 10.3 | Unknown |
| Q83D01 | RhuM | 2.3922 | CBU_0946 | 37 | 10.1 | Unknown |
| Q83DK8 | Hypothetical membrane associated protein | 2.3651 | CBU_0718 | 10.4 | 10.8 | Unknown |
| Q83C69 | Uncharacterized protein CBU_1260 | 2.3551 | CBU_1260 | 26.2 | 9.9 | Unknown |
| Q83A39 | Hypothetical exported protein | 2.2834 | CBU_2072 | 18.4 | 9.6 | Unknown |
| Q83DZ3 | LemA | 2.2574 | CBU_0545 | 22.1 | 9.5 | Unknown |
| Q83DD6 | Putative uncharacterized protein | 2.238 | CBU_0802 | 29.6 | 6.4 | Unknown |
| Q83D52 | Hypothetical exported membrane associated protein | 2.228 | CBU_0891 | 34.3 | 5 | Unknown |
| Q83AW6 | FAD dependent oxidoreductase | 2.1776 | CBU_1762 | 51.8 | 7.5 | Unknown |
| Q83DQ7 | Pyruvate dehydrogenase E1 component beta subunit | 2.1545 | CBU_0639 | 35.4 | 6.4 | Unknown |
| Q83A97 | Putative uncharacterized protein | 2.1282 | CBU_2009 | 49.4 | 9.3 | Unknown |
| Q83D49 | Cell division related protein | 2.1121 | CBU_0895 | 22.6 | 10.6 | Unknown |
| Q83F72 | Monooxygenase | 2.0864 | CBU_0076 | 46 | 9.2 | Unknown |
| Q83AP5 | Homoserine dehydrogenase | 2.0673 | CBU_1836 | 46.5 | 6.7 | Unknown |
| Q83BB8 | GatB/YqeY domain protein | 2.0571 | CBU_1594 | 16.7 | 6 | Unknown |
| Q83DQ6 | Pyruvate dehydrogenase E1 component alpha subunit | 2.0403 | CBU_0640 | 41.1 | 5 | Unknown |
| Q83FC5 | Putative pit accessory protein | 2.0008 | CBU_0015 | 26 | 7.2 | Unknown |
| Q83DV6 | Pyridine nucleotide-disulfide oxidoreductase family | 1.9967 | CBU_0586 | 132 | 6.9 | Unknown |
| Q83DA2 | Radical SAM superfamily protein | 1.9136 | CBU_0836 | 37.6 | 8.8 | Unknown |
| Q83AV6 | Probable GTP-binding protein engB | 1.8597 | CBU_1772 | 23 | 8.4 | Unknown |
| Q83A89 | Putative uncharacterized protein | 1.8531 | CBU_2018 | 22.4 | 7.1 | Unknown |
| Q83CX9 | Hypothetical cytosolic protein | 1.83 | CBU_0972 | 26.6 | 5.5 | Unknown |
| Q83C79 | Integral membrane protein | 1.8245 | CBU_1249 | 23 | 10 | Unknown |
| Q83EF5 | Hypothetical exported protein | 1.7935 | CBU_0368 | 41.1 | 9.9 | Unknown |
| Q83DI4 | LPS ABC transporter periplasmic component | 1.7918 | CBU_0747 | 19.5 | 10.5 | Unknown |
| Q83EH1 | Hpt domain protein | 1.7648 | CBU_0351 | 13.4 | 6.5 | Unknown |
| Q83B57 | Putative uncharacterized protein | 1.6995 | CBU_1658 | 41.8 | 10 | Unknown |
| Q83C43 | Putative uncharacterized protein | 1.6511 | CBU_1291 | 21.5 | 9.5 | Unknown |
| Q83BF2 | Putative uncharacterized protein | 1.6492 | CBU_1558 | 10.3 | 5.2 | Unknown |
| Q820W1 | Membrane protease family, stomatin/prohibitin homolog | 1.5686 | CBU_1482 | 28.3 | 5.4 | Unknown |
| Q83AR3 | Lysyl-tRNA synthetase | 1.5362 | CBU_1817 | 37.2 | 6.1 | Unknown |
| Q83BJ5 | Short chain dehydrogenase | 1.508 | CBU_1513 | 28.2 | 8.3 | Unknown |
| Q83A18 | UPF0301 protein CBU_2093 | 1.504 | CBU_2093 | 19.9 | 5.9 | Unknown |
| Q83AY3 | Lipoprotein | 1.4913 | CBU_1744 | 20.6 | 10.7 | Unknown |
| Q83D34 | Hypothetical membrane associated protein | 1.467 | CBU_0910 | 36.9 | 5.4 | Unknown |
| Q83EP5 | Putative reductase CBU_0270 | 1.4399 | CBU_0270 | 44.8 | 5.9 | Unknown |
| Q83AK8 | Hypothetical ATPase | 1.4259 | CBU_1877 | 49.8 | 9.2 | Unknown |
| Q83D08 | ASMA | 1.4081 | CBU_0939 | 54.9 | 10.7 | Unknown |
| Q83BE4 | UPF0082 protein CBU_1566 | 1.3716 | CBU_1566 | 26.5 | 4.6 | Unknown |
| Q83FA3 | Nucleoside-triphosphatase | 1.348 | CBU_0043 | 21.7 | 4.4 | Unknown |
| Q83FB2 | 1-acyl-sn-glycerol-3-phosphate acyltransferase | 1.3447 | CBU_0029 | 28 | 10.4 | Unknown |
| Q83DZ1 | Tetratricopeptide repeat family protein | 1.3352 | CBU_0547 | 64.4 | 7.4 | Unknown |
| Q83CC4 | Ferredoxin-dependent glutamate synthase | 1.3227 | CBU_1203 | 61.8 | 7.9 | Unknown |
| Q83F97 | Putative uncharacterized protein | 1.282 | CBU_0051 | 21.2 | 9.5 | Unknown |
| Q93N44 | Sulfotransferase | 1.2313 | CBU_0699 | 61.6 | 6 | Unknown |
| Q83CA4 | RmuC family protein | 1.21 | CBU_1224 | 51.5 | 7.3 | Unknown |
| Q83BX7 | ABC transporter-associated protein | 1.1227 | CBU_1360 | 53.5 | 6.5 | Unknown |
| Q83BK3 | GTP-binding protein lepA | 1.0911 | CBU_1505 | 67.8 | 7.9 | Unknown |
| Q83BN5 | Putative uncharacterized protein | 1.0892 | CBU_1468 | 126.8 | 10.2 | Unknown |
| Q83BW5 | Hypothetical membrane associated protein | 1.0724 | CBU_1372 | 21.7 | 9.2 | Unknown |
| Q83DM1 | Methyltransferase | 1.0401 | CBU_0682 | 24.6 | 5.1 | Unknown |
| Q83BI7 | Rhodanese-related sulfurtransferase | 0.989 | CBU_1521 | 16.2 | 10.4 | Unknown |
| Q83CN2 | Putative uncharacterized protein | 0.9659 | CBU_1079 | 23.5 | 5.9 | Unknown |
| Q839Z7 | Plasmid replication initiation protein | 0.9201 | CBU_A0039 | 49.8 | 9.8 | Unknown |
| Q83AZ4 | D-3-phosphoglycerate dehydrogenase | 0.8944 | CBU_1732 | 42.8 | 7.0 | Unknown |
| Q83EK8 | Outer membrane protein P1 | 0.8515 | CBU_0311 | 23.9 | 9.2 | Unknown |
| Q83EV0 | Putative uncharacterized protein | 0.8427 | CBU_0209 | 37.7 | 5.5 | Unknown |
| Q83BS7 | 17 kDa common-antigen | 0.7856 | CBU_1425 | 15.9 | 10.2 | Unknown |
| Q83F98 | Hypothetical cytosolic protein | 0.785 | CBU_0050 | 22.7 | 8.4 | Unknown |
| Q83AQ3 | Putative uncharacterized protein | 0.7654 | CBU_1828 | 43.6 | 6.3 | Unknown |
| Q83C83 | GTP-binding protein engA | 0.7588 | CBU_1245 | 49.7 | 9.6 | Unknown |
| Q83CD7 | Glycine-rich RNA-binding protein | 0.7527 | CBU_1183 | 11.2 | 9.5 | Unknown |
| Q83BX9 | SufD | 0.7387 | CBU_1358 | 48.0 | 6.5 | Unknown |
| Q83DY7 | Iojap protein family | 0.6791 | CBU_0552 | 13.1 | 4.6 | Unknown |
| Q83F80 | Putative uncharacterized protein | 0.6727 | CBU_0068 | 9.3 | 10.8 | Unknown |
| Q83C81 | Putative uncharacterized protein | 0.6466 | CBU_1247 | 23.7 | 9.7 | Unknown |
| Q83A93 | Putative uncharacterized protein | 0.5813 | CBU_2013 | 40.7 | 6.8 | Unknown |
| Q83DB0 | NAD-dependent oxidoreductase | 0.5543 | CBU_0828 | 57.5 | 7.3 | Unknown |
| Q83DG3 | PhnB | 0.5486 | CBU_0773 | 17.3 | 6.3 | Unknown |
| Q83DK1 | Hypothetical exported protein | 0.5236 | CBU_0729 | 33.9 | 9.7 | Unknown |
| Q83BF9 | Phosphoenolpyruvate-protein phosphotransferase | 70.1508 | CBU_1550 | 84 | 4.8 | Transport and binding |
| Q820W5 | Hpr(Ser) kinase | 12.7783 | CBU_0744 | 26.4 | 6.9 | Transport and binding |
| Q83AW2 | Ferrous iron transport protein B | 12.6398 | CBU_1766 | 89.9 | 10 | Transport and binding |
| Q83DI7 | Phosphocarrier protein HPr | 8.1044 | CBU_0743 | 10 | 8.4 | Transport and binding |
| Q83E49 | Arginine-binding protein | 6.2159 | CBU_0482 | 29.6 | 9.8 | Transport and binding |
| Q83EY0 | Glycine betaine transport ATP-binding protein | 4.8375 | CBU_0178 | 28.5 | 9.4 | Transport and binding |
| Q83F92 | Type I secretion outer membrane protein | 3.8645 | CBU_0056 | 68.6 | 10 | Transport and binding |
| Q83BX5 | Cobalt-zinc-cadmium resistance protein | 3.3858 | CBU_1362 | 41.6 | 6.8 | Transport and binding |
| Q83AS1 | ABC transporter ATP-binding protein | 3.3718 | CBU_1809 | 24.4 | 10.1 | Transport and binding |
| Q83D84 | Lipid A export ATP-binding/permease protein msbA | 2.6428 | CBU_0856 | 65.9 | 9.8 | Transport and binding |
| Q83BX8 | ATP-dependent transporter | 2.181 | CBU_1359 | 27 | 5.6 | Transport and binding |
| Q83F42 | Methionine-binding protein | 2.0362 | CBU_0109 | 28.8 | 9.8 | Transport and binding |
| Q83D12 | ABC transporter ATP-binding protein | 1.7875 | CBU_0934 | 34.5 | 8.9 | Transport and binding |
| Q83AS2 | Export ABC transporter permease protein | 1.6339 | CBU_1808 | 44.3 | 10.2 | Transport and binding |
| Q83CM0 | Acriflavin resistance periplasmic protein | 1.6003 | CBU_1094 | 41 | 9.9 | Transport and binding |
| Q83E50 | Arginine transport ATP-binding protein | 1.3861 | CBU_0481 | 27.8 | 8.4 | Transport and binding |
| Q83BK1 | Copper-exporting ATPase | 1.3561 | CBU_1507 | 79.3 | 9.3 | Transport and binding |
| Q83DK2 | ABC transporter ATP-binding protein | 1.0368 | CBU_0728 | 29.8 | 7.3 | Transport and binding |
| Q83D38 | Transporter, MFS superfamily | 1.0291 | CBU_0906 | 47.7 | 8.8 | Transport and binding |
| Q83EP3 | Transporter, MFS superfamily | 0.9963 | CBU_0272 | 50.7 | 9.9 | Transport and binding |
| Q83DC6 | Na+ driven multidrug efflux pump | 0.8011 | CBU_0812 | 50.3 | 10.0 | Transport and binding |
| Q83BZ0 | Glutamate/gamma-aminobutyrate antiporter | 0.7783 | CBU_1347 | 51.6 | 10.2 | Transport and binding |
| Q83DI5 | LPS ABC transporter ATP-binding protein | 0.7514 | CBU_0746 | 27.8 | 9.1 | Transport and binding |
| Q83AE9 | ABC transporter ATP-binding protein | 0.4914 | CBU_1952 | 59.5 | 8.0 | Transport and binding |
| Q83DI6 | Ribosome-associated factor Y | 7.5435 | CBU_0745 | 11 | 9.8 | Translation and protein biosynthesis |
| Q83BB0 | Ribosomal protein S6 modification protein | 5.7945 | CBU_1602 | 33.1 | 10.3 | Translation and protein biosynthesis |
| Q83DY1 | Leucyl-tRNA synthetase | 3.0159 | CBU_0559 | 94.2 | 7.8 | Translation and protein biosynthesis |
| Q83BJ9 | tRNA(Ile)-lysidine synthase | 2.4108 | CBU_1509 | 51.3 | 8.9 | Translation and protein biosynthesis |
| Q4AAY1 | Pseudouridine synthase | 2.3509 | CBU_0487 | 34.6 | 10.6 | Translation and protein biosynthesis |
| Q4AAX7 | tRNA pseudouridine synthase B | 2.2582 | CBU_1430 | 34.6 | 9.9 | Translation and protein biosynthesis |
| Q83A98 | Arginyl-tRNA synthetase | 2.0949 | CBU_2008 | 66.7 | 6.5 | Translation and protein biosynthesis |
| Q83BM9 | Glutamyl-tRNA(Gln) amidotransferase subunit A | 2.0739 | CBU_1474 | 52.5 | 6.5 | Translation and protein biosynthesis |
| O85388 | 30S ribosomal protein S3 | 1.9918 | CBU_0244 | 25.6 | 10.6 | Translation and protein biosynthesis |
| Q83AA8 | Methionyl-tRNA formyltransferase | 1.8865 | CBU_1997 | 34.2 | 8 | Translation and protein biosynthesis |
| Q83AR4 | Elongation factor P | 1.8526 | CBU_1816 | 21 | 5.7 | Translation and protein biosynthesis |
| Q83EC9 | Isoleucyl-tRNA synthetase | 1.7882 | CBU_0396 | 106.1 | 6.2 | Translation and protein biosynthesis |
| Q83CQ6 | Alanyl-tRNA synthetase | 1.7647 | CBU_1052 | 97.6 | 6.7 | Translation and protein biosynthesis |
| Q83EX7 | Tyrosyl-tRNA synthetase | 1.6251 | CBU_0181 | 45.8 | 6.6 | Translation and protein biosynthesis |
| Q83ES6 | Elongation factor Tu | 1.5743 | CBU_0236 | 43.5 | 5.1 | Translation and protein biosynthesis |
| Q83BS1 | Translation initiation factor IF-2 | 1.5479 | CBU_1432 | 88.4 | 6.9 | Translation and protein biosynthesis |
| Q83EM4 | 50S ribosomal protein L28 | 1.5351 | CBU_0291 | 9.2 | 11.9 | Translation and protein biosynthesis |
| Q83F67 | Prolyl-tRNA synthetase | 1.5346 | CBU_0081 | 63.5 | 5.9 | Translation and protein biosynthesis |
| Q83ER3 | SSU ribosomal protein S14P | 1.5145 | CBU_0251 | 11.5 | 11.6 | Translation and protein biosynthesis |
| P45651 | Glycyl-tRNA synthetase beta subunit | 1.4872 | CBU_1914 | 77.9 | 8 | Translation and protein biosynthesis |
| Q83ES5 | 30S ribosomal protein S10 | 1.482 | CBU_0237 | 12.5 | 10.2 | Translation and protein biosynthesis |
| Q83ES0 | 30S ribosomal protein S19 | 1.479 | CBU_0242 | 10.8 | 11.1 | Translation and protein biosynthesis |
| Q83E85 | 50S ribosomal protein L19 | 1.4113 | CBU_0442 | 13.2 | 11.4 | Translation and protein biosynthesis |
| Q83BV4 | Ribosome-recycling factor | 1.3988 | CBU_1383 | 20.9 | 6.8 | Translation and protein biosynthesis |
| Q83BE5 | Aspartyl-tRNA synthetase | 1.3895 | CBU_1565 | 66.7 | 6.3 | Translation and protein biosynthesis |
| Q9X5U9 | Elongation factor Ts | 1.3618 | CBU_1385 | 31.8 | 5.8 | Translation and protein biosynthesis |
| P59753 | 30S ribosomal protein S13 | 1.3466 | CBU_0260 | 13.3 | 11.5 | Translation and protein biosynthesis |
| Q83ER7 | 30S ribosomal protein S17 | 1.321 | CBU_0247 | 10.3 | 10.9 | Translation and protein biosynthesis |
| Q83ED6 | 30S ribosomal protein S20 | 1.3012 | CBU_0389 | 9.9 | 12.2 | Translation and protein biosynthesis |
| Q820W2 | tRNA-specific 2-thiouridylase mnmA | 1.288 | CBU_1147 | 40.8 | 7.3 | Translation and protein biosynthesis |
| Q83C80 | Histidyl-tRNA synthetase | 1.2675 | CBU_1248 | 47.8 | 5.9 | Translation and protein biosynthesis |
| Q4AAX8 | Pseudouridine synthase | 1.2602 | CBU_1059 | 27.8 | 10.4 | Translation and protein biosynthesis |
| Q83EQ3 | 30S ribosomal protein S4 | 1.2446 | CBU_0262 | 23.7 | 10.5 | Translation and protein biosynthesis |
| Q83E09 | 30S ribosomal protein S1 | 1.2414 | CBU_0528 | 62.1 | 5 | Translation and protein biosynthesis |
| Q83E97 | Lysyl-tRNA synthetase | 1.2362 | CBU_0430 | 57.7 | 6.4 | Translation and protein biosynthesis |
| Q83EQ9 | 30S ribosomal protein S5 | 1.2357 | CBU_0255 | 17.4 | 10.5 | Translation and protein biosynthesis |
| Q9X5U8 | 30S ribosomal protein S2 | 1.2186 | CBU_1386 | 35.3 | 9.3 | Translation and protein biosynthesis |
| Q83ES8 | 30S ribosomal protein S7 | 1.2178 | CBU_0234 | 21.2 | 10.9 | Translation and protein biosynthesis |
| Q83ER1 | 50S ribosomal protein L6 | 1.157 | CBU_0253 | 19.1 | 10.8 | Translation and protein biosynthesis |
| Q83D75 | 30S ribosomal protein S18 | 1.1513 | CBU_0865 | 8.5 | 10 | Translation and protein biosynthesis |
| Q83C11 | Translation initiation factor IF-3 | 1.1425 | CBU_1325 | 19.4 | 10.6 | Translation and protein biosynthesis |
| Q83EQ4 | 30S ribosomal protein S11 | 1.1217 | CBU_0261 | 13.5 | 11.8 | Translation and protein biosynthesis |
| Q83ER9 | 50S ribosomal protein L16 | 1.0995 | CBU_0245 | 15.4 | 11.7 | Translation and protein biosynthesis |
| Q83ER2 | 30S ribosomal protein S8 | 1.0613 | CBU_0252 | 14.5 | 9.8 | Translation and protein biosynthesis |
| Q83E83 | 30S ribosomal protein S16 | 1.0481 | CBU_0445 | 15.5 | 10.5 | Translation and protein biosynthesis |
| Q83ES3 | 50S ribosomal protein L4 | 1.028 | CBU_0239 | 22.3 | 10.6 | Translation and protein biosynthesis |
| O85387 | 50S ribosomal protein L22 | 1.0219 | CBU_0243 | 12.5 | 10.4 | Translation and protein biosynthesis |
| Q83ER4 | 50S ribosomal protein L5 | 1.0163 | CBU_0250 | 20.7 | 10.3 | Translation and protein biosynthesis |
| Q83ES4 | 50S ribosomal protein L3 | 0.9908 | CBU_0238 | 23.5 | 10.5 | Translation and protein biosynthesis |
| Q83D76 | 30S ribosomal protein S6 | 0.9848 | CBU_0864 | 14.5 | 7.9 | Translation and protein biosynthesis |
| Q83EQ7 | 50S ribosomal protein L15 | 0.9809 | CBU_0257 | 15.2 | 11.8 | Translation and protein biosynthesis |
| Q83AP1 | 50S ribosomal protein L25 | 0.9782 | CBU_1840 | 26.5 | 4.7 | Translation and protein biosynthesis |
| Q83EE0 | 50S ribosomal protein L21 | 0.9782 | CBU_0385 | 12.8 | 10.4 | Translation and protein biosynthesis |
| Q83C14 | Phenylalanyl-tRNA synthetase alpha chain | 0.9468 | CBU_1322 | 37.8 | 7.6 | Translation and protein biosynthesis |
| Q83AX8 | 50S ribosomal protein L13 | 0.944 | CBU_1749 | 15.8 | 10.6 | Translation and protein biosynthesis |
| Q83D88 | 30S ribosomal protein S15 | 0.9363 | CBU_0851 | 10.3 | 11.1 | Translation and protein biosynthesis |
| Q83C15 | Phenylalanyl-tRNA synthetase beta chain | 0.9356 | CBU_1321 | 88.4 | 5.7 | Translation and protein biosynthesis |
| Q83ER8 | 50S ribosomal protein L29 | 0.8856 | CBU_0246 | 7.6 | 11.0 | Translation and protein biosynthesis |
| Q83ES1 | 50S ribosomal protein L2 | 0.8813 | CBU_0241 | 30.4 | 11.7 | Translation and protein biosynthesis |
| Q83D39 | 50S ribosomal protein L31 | 0.8717 | CBU_0905 | 9.0 | 9.6 | Translation and protein biosynthesis |
| Q83ET4 | 50S ribosomal protein L11 | 0.8715 | CBU_0226 | 15.5 | 10.3 | Translation and protein biosynthesis |
| Q83D73 | 50S ribosomal protein L9 | 0.8573 | CBU_0867 | 16.5 | 6.5 | Translation and protein biosynthesis |
| Q83EM5 | 50S ribosomal protein L33 | 0.8489 | CBU_0290 | 8.0 | 10.9 | Translation and protein biosynthesis |
| Q83ET3 | 50S ribosomal protein L1 | 0.847 | CBU_0227 | 24.7 | 10.3 | Translation and protein biosynthesis |
| Q83ER5 | 50S ribosomal protein L24 | 0.8383 | CBU_0249 | 11.7 | 10.8 | Translation and protein biosynthesis |
| P47849 | Peptide chain release factor 1 | 0.8 | CBU_1965 | 40.7 | 5.1 | Translation and protein biosynthesis |
| Q83ES7 | Elongation factor G | 0.7742 | CBU_0235 | 77.7 | 4.7 | Translation and protein biosynthesis |
| Q83ET2 | 50S ribosomal protein L10 | 0.7646 | CBU_0228 | 19.1 | 9.8 | Translation and protein biosynthesis |
| Q83DD0 | Valyl-tRNA synthetase | 0.7644 | CBU_0808 | 106.6 | 8.7 | Translation and protein biosynthesis |
| P0C8S3 | 50S ribosomal protein L7/L12 | 0.7599 | CBU_0229 | 13.2 | 4.3 | Translation and protein biosynthesis |
| Q83ER0 | 50S ribosomal protein L18 | 0.7195 | CBU_0254 | 13.1 | 11.4 | Translation and protein biosynthesis |
| Q83EQ8 | 50S ribosomal protein L30 | 0.6894 | CBU_0256 | 7.2 | 9.7 | Translation and protein biosynthesis |
| P94612 | tRNA modification GTPase mnmE | 0.6081 | CBU_1922 | 52.0 | 6.5 | Translation and protein biosynthesis |
| Q83DC7 | Peptide chain release factor 3 | 0.5982 | CBU_0811 | 59.4 | 6.7 | Translation and protein biosynthesis |
| Q83B24 | Methionyl-tRNA synthetase | 0.5824 | CBU_1695 | 62.2 | 6.6 | Translation and protein biosynthesis |
| Q83C13 | 50S ribosomal protein L20 | 0.526 | CBU_1323 | 13.4 | 12.0 | Translation and protein biosynthesis |
| Q83E41 | 50S ribosomal protein L32 | 0.4857 | CBU_0491 | 7.4 | 10.5 | Translation and protein biosynthesis |
| Q83B16 | Ribonuclease T | 3.0242 | CBU_1704 | 24.7 | 8 | Transcription |
| Q83E45 | Ribonuclease E | 2.752 | CBU_0486 | 80.3 | 9.7 | Transcription |
| Q83EL5 | Translation initiation inhibitor | 2.714 | CBU_0304 | 14 | 6.5 | Transcription |
| Q83EM9 | Poly(A) polymerase | 2.4761 | CBU_0286 | 52.4 | 10.7 | Transcription |
| Q83CM3 | Ribonuclease R | 2.3353 | CBU_1091 | 85.1 | 9.5 | Transcription |
| Q83BB6 | RNA polymerase sigma factor rpoD | 2.2083 | CBU_1596 | 79.5 | 9.7 | Transcription |
| Q83BT5 | N utilization substance protein B homolog | 2.0898 | CBU_1417 | 15.8 | 5.9 | Transcription |
| Q83EQ2 | DNA-directed RNA polymerase subunit alpha | 1.8113 | CBU_0263 | 35.5 | 5.6 | Transcription |
| Q83ET0 | DNA-directed RNA polymerase subunit beta | 1.4558 | CBU_0232 | 157.1 | 7.8 | Transcription |
| Q83A25 | Transcription termination factor rho | 1.4499 | CBU_2086 | 46.8 | 6.6 | Transcription |
| P0C8S4 | DNA-directed RNA polymerase subunit beta | 1.4405 | CBU_0231 | 153.6 | 5.9 | Transcription |
| Q83C51 | Transcription elongation factor | 1.2368 | CBU_1280 | 17.6 | 4.4 | Transcription |
| Q83BZ8 | Ribonuclease HII | 1.1242 | CBU_1339 | 22.3 | 7.5 | Transcription |
| Q83D87 | Polyribonucleotide nucleotidyltransferase | 1.0829 | CBU_0852 | 76.3 | 5.1 | Transcription |
| Q83BS0 | Transcription elongation protein nusA | 1.0526 | CBU_1433 | 56.2 | 4.3 | Transcription |
| Q820V9 | CCA-adding enzyme | 0.9691 | CBU_1827 | 42.4 | 8.9 | Transcription |
| Q83ET5 | Transcription antitermination protein nusG | 0.7608 | CBU_0225 | 20.0 | 6.8 | Transcription |
| Q83AB3 | [RelE](http://www.ebi.ac.uk/cgi-bin/dbfetch?db=emblcds&id=AAO91481) | 4.4912 | CBU_1992 | 11.1 | 9.6 | Toxin production and resistance |
| Q93N52 | Methyltransferase | 2.3327 | CBU_0691 | 44.2 | 6 | Toxin production and resistance |
| Q83F59 | Protein tolB | 2.1271 | CBU_0090 | 47.7 | 8.9 | Toxin production and resistance |
| Q83DD1 | Beta-lactamase family protein | 1.2964 | CBU_0807 | 42.6 | 9.3 | Toxin production and resistance |
| Q83DE0 | Multidrug resistance protein A | 1.0089 | CBU_0798 | 37.0 | 9.9 | Toxin production and resistance |
| Q83DD4 | Acriflavin resistance plasma membrane protein | 0.9246 | CBU_0804 | 110.0 | 8.5 | Toxin production and resistance |
| Q83DH8 | Acriflavin resistance plasma membrane protein | 0.683 | CBU_0753 | 110.9 | 7.9 | Toxin production and resistance |
| P39917 | Outer-membrane lipoprotein carrier protein | 3.3784 | CBU_1190 | 23.8 | 10.7 | Secretion and trafficking |
| Q83E79 | Signal recognition particle, subunit FFH/SRP54 | 1.3656 | CBU_0450 | 50.9 | 10.4 | Secretion and trafficking |
| Q83DJ3 | Trigger factor | 1.3218 | CBU_0737 | 50.2 | 5 | Secretion and trafficking |
| Q83F06 | Protein translocase subunit secA | 1.2347 | CBU_0147 | 103.8 | 5.6 | Secretion and trafficking |
| Q83CV2 | Lipoprotein-releasing system ATP-binding protein lolD | 1.1307 | CBU_1000 | 25.8 | 8.9 | Secretion and trafficking |
| Q83E51 | Arginine repressor | 5.2631 | CBU_0480 | 17.7 | 10.1 | Regulatory function |
| Q83DG1 | Transcriptional regulator, GntR family | 4.4069 | CBU_0775 | 13.3 | 10.2 | Regulatory function |
| B5U8Q1 | Nucleotide-sugar aminotransferase | 4.1038 | CBU_0696 | 43.1 | 6.5 | Regulatory function |
| Q83DL2 | Response regulator | 3.8302 | CBU_0712 | 23.6 | 9.5 | Regulatory function |
| Q83BD2 | Trp repressor binding protein | 3.5741 | CBU_1579 | 21.1 | 7.6 | Regulatory function |
| Q83EF6 | Phosphate regulon transcriptional regulatory protein | 3.4932 | CBU_0367 | 28.8 | 7.9 | Regulatory function |
| Q83CA1 | Transcriptional regulatory protein | 3.4239 | CBU_1227 | 25.2 | 8.4 | Regulatory function |
| Q83DC0 | Transcriptional regulator, TetR family | 2.5002 | CBU_0818 | 22.1 | 9.8 | Regulatory function |
| Q83BT4 | Transcriptional repressor nrdR | 2.2416 | CBU_1418 | 18.5 | 9 | Regulatory function |
| Q83CZ5 | Response regulator | 2.2234 | CBU_0955 | 24.1 | 10.1 | Regulatory function |
| Q83AW7 | Sensor protein | 1.9201 | CBU_1761 | 74.5 | 9.3 | Regulatory function |
| Q83EF7 | Sensor protein | 1.6935 | CBU_0366 | 28.9 | 9.4 | Regulatory function |
| Q93N46 | dTDP-4-dehydro-6-deoxy-D-glucose 4-aminotransferase | 1.2945 | CBU_0697 | 43.5 | 6.9 | Regulatory function |
| Q83DR2 | Sensor protein | 1.2332 | CBU_0634 | 62.3 | 6.7 | Regulatory function |
| Q83C33 | Ferric uptake regulation protein | 1.1708 | CBU_1301 | 17.7 | 6.7 | Regulatory function |
| Q83AA0 | Two-component response regulator | 1.0613 | CBU_2006 | 27.3 | 6.9 | Regulatory function |
| Q83C17 | Transcriptional regulator, MerR family | 0.4553 | CBU_1319 | 15.0 | 9.9 | Regulatory function |
| Q83CE9 | Small heat shock protein | 118.9224 | CBU_1169 | 17.2 | 5.7 | Protein fate |
| P19421 | 60 kDa chaperonin | 6.7186 | CBU_1718 | 58.2 | 4.8 | Protein fate |
| P19422 | 10 kDa chaperonin | 4.4951 | CBU_1719 | 10.5 | 4.9 | Protein fate |
| Q83AI5 | Non-proteolytic protein, peptidase family M16 | 4.2279 | CBU_1901 | 48.1 | 9.5 | Protein fate |
| Q83AK6 | Peptide deformylase 2 | 4.1899 | CBU_1879 | 23.9 | 5.3 | Protein fate |
| Q83AC4 | Peptidyl-prolyl cis-trans isomerase | 4.1292 | CBU_1980 | 33.2 | 10.4 | Protein fate |
| Q83CD0 | ATP-dependent clp protease ATP-binding subunit | 3.3642 | CBU_1196 | 84.3 | 7.3 | Protein fate |
| Q83CH4 | Protein translocase subunit | 3.2666 | CBU_1141 | 33.5 | 10.3 | Protein fate |
| Q83BH0 | Carboxy-terminal processing protease | 3.1062 | CBU_1538 | 49.6 | 9.3 | Protein fate |
| O87712 | Chaperone protein dnaK | 3.0816 | CBU_1290 | 70.7 | 4.8 | Protein fate |
| Q83DH6 | Endopeptidase | 2.5209 | CBU_0755 | 47.7 | 9.1 | Protein fate |
| Q83EI2 | Membrane alanine aminopeptidase | 2.4543 | CBU_0338 | 100.5 | 6.5 | Protein fate |
| Q83F55 | Chaperone protein clpB | 2.4079 | CBU_0094 | 96.3 | 5.7 | Protein fate |
| Q83F46 | Peptidase, M20A family | 2.3993 | CBU_0103 | 52.8 | 5.2 | Protein fate |
| Q83A94 | ATP-dependent hsl protease ATP-binding subunit hslU | 2.1856 | CBU_2012 | 50.1 | 5.3 | Protein fate |
| Q83C41 | Protein grpE | 2.1529 | CBU_1293 | 22.9 | 5.2 | Protein fate |
| Q83AI3 | Cell division protein | 2.1007 | CBU_1903 | 35.4 | 9.8 | Protein fate |
| Q83DJ0 | ATP-dependent endopeptidase | 1.9416 | CBU_0740 | 91.6 | 9.2 | Protein fate |
| Q83CH2 | Protein translocase subunit | 1.9115 | CBU_1143 | 12.5 | 10.1 | Protein fate |
| Q83AI4 | Peptidase, M16 family | 1.8937 | CBU_1902 | 51.9 | 6.7 | Protein fate |
| Q83EL0 | Chaperone protein htpG | 1.7285 | CBU_0309 | 72.7 | 4.9 | Protein fate |
| Q83A95 | ATP-dependent protease hslV | 1.7142 | CBU_2011 | 19.5 | 9.8 | Protein fate |
| P59650 | Deoxyhypusine synthase-like protein | 1.7133 | CBU_0721 | 39.5 | 5.5 | Protein fate |
| Q83CJ2 | Curved DNA-binding protein | 1.6377 | CBU_1123 | 34.9 | 7.7 | Protein fate |
| Q83EG9 | Non-proteolytic protein, peptidase family S49 | 1.6185 | CBU_0353 | 38.3 | 10 | Protein fate |
| Q83FA6 | Oligopeptidase A | 1.5498 | CBU_0039 | 78.2 | 6.4 | Protein fate |
| Q83C28 | Peptide methionine sulfoxide reductase | 1.5071 | CBU_1306 | 32.8 | 7.2 | Protein fate |
| Q83BT6 | Uncharacterized HTH-type transcriptional regulator CBU_1416 | 1.5063 | CBU_1416 | 24.2 | 7.5 | Protein fate |
| Q820B4 | Apolipoprotein N-acyltransferase | 1.1079 | CBU_0564 | 54.6 | 9.7 | Protein fate |
| Q83AB8 | Signal peptide peptidase | 1.0715 | CBU_1986 | 35.1 | 7.7 | Protein fate |
| Q83CV9 | Peptide deformylase 1 | 0.9339 | CBU_0993 | 19.3 | 8.3 | Protein fate |
| Q83B02 | Thiol:disulfide interchange protein | 0.8871 | CBU_1723 | 63.6 | 9.9 | Protein fate |
| Q83CL5 | Signal peptidase I | 0.113 | CBU_1099 | 30.1 | 10.1 | Protein fate |
| Q83B86 | IcmE | 6.2988 | CBU_1627 | 105.9 | 9.4 | Pathogenesis |
| P51752 | Peptidyl-prolyl cis-trans isomerase Mi | 4.7426 | CBU_0630 | 25.5 | 10.4 | Pathogenesis |
| Q83B81 | IcmO | 4.5269 | CBU_1632 | 89.2 | 6.9 | Pathogenesis |
| Q83B73 | IcmS | 2.6934 | CBU_1642 | 12.3 | 4.8 | Pathogenesis |
| Q83B84 | IcmL | 2.5722 | CBU_1629 | 24.9 | 8.4 | Pathogenesis |
| Q83B65 | IcmW | 2.4767 | CBU_1650 | 16.9 | 5.2 | Pathogenesis |
| Q83B79 | IcmQ | 2.3323 | CBU_1634 | 27.1 | 8 | Pathogenesis |
| Q83B87 | IcmG | 2.2639 | CBU_1626 | 26.5 | 5.3 | Pathogenesis |
| Q83B91 | IcmB | 2.1994 | CBU_1622 | 112.4 | 6.3 | Pathogenesis |
| Q83B71 | DotC | 0.9087 | CBU_1644 | 30.2 | 8.8 | Pathogenesis |
| Q83FC4 | Xanthosine phosphorylase | 20.3568 | CBU_0016 | 30.1 | 6.8 | Nucleotide and Nucleoside metabolism |
| Q83EJ4 | Phosphoribosylamine--glycine ligase | 4.9726 | CBU_0326 | 46.1 | 6.6 | Nucleotide and Nucleoside metabolism |
| Q83CA8 | Phosphoribosylaminoimidazole-succinocarboxamide synthase | 3.6042 | CBU_1220 | 27.4 | 6.7 | Nucleotide and Nucleoside metabolism |
| Q45918 | Orotate phosphoribosyltransferase | 3.2074 | CBU_0296 | 23.4 | 7.1 | Nucleotide and Nucleoside metabolism |
| Q83D47 | Amidophosphoribosyltransferase | 2.8982 | CBU_0897 | 55.9 | 6.4 | Nucleotide and Nucleoside metabolism |
| Q83A16 | Aspartate carbamoyltransferase | 2.6272 | CBU_2095 | 34.9 | 9.4 | Nucleotide and Nucleoside metabolism |
| Q83BV3 | Uridylate kinase | 2.1711 | CBU_1384 | 26.3 | 9.3 | Nucleotide and Nucleoside metabolism |
| Q83CV4 | Adenylosuccinate synthetase | 2.1327 | CBU_0998 | 47.9 | 6.5 | Nucleotide and Nucleoside metabolism |
| Q83DB4 | Adenylosuccinate lyase | 1.9702 | CBU_0824 | 52.5 | 7.7 | Nucleotide and Nucleoside metabolism |
| Q83CN6 | Hypoxanthine-guanine phosphoribosyltransferase | 1.8992 | CBU_1074 | 20.4 | 5.8 | Nucleotide and Nucleoside metabolism |
| Q83EI4 | Phosphoribosylaminoimidazolecarboxamide formyltransferase | 1.7227 | CBU_0336 | 57.4 | 6.9 | Nucleotide and Nucleoside metabolism |
| Q83DR5 | Ribose-phosphate pyrophosphokinase | 1.7022 | CBU_0631 | 143.3 | 6.6 | Nucleotide and Nucleoside metabolism |
| Q83AQ1 | Ribose-phosphate pyrophosphokinase | 1.7011 | CBU_1830 | 35.2 | 6.1 | Nucleotide and Nucleoside metabolism |
| Q83BZ6 | GMP synthase [glutamine-hydrolyzing] | 1.6378 | CBU_1341 | 58.6 | 6.7 | Nucleotide and Nucleoside metabolism |
| Q83E75 | Adenylate kinase | 1.6361 | CBU_0454 | 25.8 | 6.8 | Nucleotide and Nucleoside metabolism |
| Q83AZ0 | Phosphoribosylformylglycinamidine cyclo-ligase | 1.5998 | CBU_1736 | 38.2 | 7.1 | Nucleotide and Nucleoside metabolism |
| Q83B36 | CTP synthase | 1.4776 | CBU_1682 | 61 | 6.1 | Nucleotide and Nucleoside metabolism |
| Q83BZ5 | Inosine-5'-monophosphate dehydrogenase | 1.4567 | CBU_1342 | 52.7 | 8.2 | Nucleotide and Nucleoside metabolism |
| Q83D69 | Uridine kinase | 1.4264 | CBU_0872 | 24.6 | 6.8 | Nucleotide and Nucleoside metabolism |
| Q83C71 | Nucleoside diphosphate kinase | 1.2518 | CBU_1258 | 15.8 | 6.9 | Nucleotide and Nucleoside metabolism |
| Q83C50 | Carbamoyl-phosphate synthase large chain | 0.7995 | CBU_1281 | 118.5 | 5.2 | Nucleotide and Nucleoside metabolism |
| Q83BF6 | Ribonucleoside-diphosphate reductase | 0.5913 | CBU_1553 | 106.4 | 6.5 | Nucleotide and Nucleoside metabolism |
| Q83AC3 | 4-hydroxythreonine-4-phosphate dehydrogenase | 6.3751 | CBU_1981 | 32.6 | 7.6 | Multi-biosynthetic pathways |
| Q83DE3 | GTP cyclohydrolase 1 | 3.5782 | CBU_0795 | 20.7 | 9.4 | Multi-biosynthetic pathways |
| Q83BL1 | Pyridoxine 5'-phosphate synthase | 3.3597 | CBU_1494 | 26 | 5.7 | Multi-biosynthetic pathways |
| Q83DP8 | 6,7-dimethyl-8-ribityllumazine synthase | 3.3473 | CBU_0648 | 16.6 | 7.3 | Multi-biosynthetic pathways |
| Q83AB2 | Dihydrofolate reductase | 3.289 | CBU_1993 | 18.6 | 9 | Multi-biosynthetic pathways |
| Q83BS8 | Delta-aminolevulinic acid dehydratase | 3.2552 | CBU_1424 | 36.8 | 7.6 | Multi-biosynthetic pathways |
| Q83DQ3 | Diaminohydroxyphosphoribosylaminopyrimidine deaminase | 2.5984 | CBU_0643 | 39.6 | 8.3 | Multi-biosynthetic pathways |
| Q83EK7 | Bifunctional protein folD | 2.1578 | CBU_0312 | 30.8 | 8.3 | Multi-biosynthetic pathways |
| Q83A37 | Porphobilinogen deaminase | 2.1429 | CBU_2074 | 33.9 | 8.5 | Multi-biosynthetic pathways |
| Q83A31 | Uroporphyrin-III C-methyltransferase | 1.9958 | CBU_2080 | 35.2 | 7.4 | Multi-biosynthetic pathways |
| Q83CS0 | Nicotinate phosphoribosyltransferase | 1.8698 | CBU_1035 | 52.8 | 9.5 | Multi-biosynthetic pathways |
| Q83DQ0 | Riboflavin synthase alpha chain | 1.4049 | CBU_0646 | 22.4 | 6.3 | Multi-biosynthetic pathways |
| Q83CD9 | Thiamine biosynthesis protein thiI | 1.3937 | CBU_1181 | 44.6 | 9.3 | Multi-biosynthetic pathways |
| Q83EV9 | Pantothenate kinase | 1.144 | CBU_0199 | 36.9 | 9.5 | Multi-biosynthetic pathways |
| Q83DP9 | GTP cyclohydrolase II | 1.0019 | CBU_0647 | 44.8 | 6.2 | Multi-biosynthetic pathways |
| Q83DV4 | Quinolinate synthetase A | 0.9204 | CBU_0588 | 36.0 | 5.1 | Multi-biosynthetic pathways |
| Q83BT7 | Thiamine-monophosphate kinase | 0.833 | CBU_1415 | 37.2 | 6.1 | Multi-biosynthetic pathways |
| Q83BV5 | Undecaprenyl pyrophosphate synthetase | 0.7965 | CBU_1382 | 27.6 | 9.3 | Multi-biosynthetic pathways |
| Q83CI6 | Cysteine desulfurase | 0.5597 | CBU_1129 | 43.0 | 7.8 | Multi-biosynthetic pathways |
| Q83EA2 | 3-methyl-2-oxobutanoate hydroxymethyltransferase | 0.3773 | CBU_0424 | 28.9 | 7.5 | Multi-biosynthetic pathways |
| Q83CA3 | 1,3,4,6-tetrachloro-1,4-cyclohexadiene hydrolase | 4.4514 | CBU_1225 | 33.7 | 7.2 | Intermediary metabolism and other metabolic pathways |
| Q83DU3 | 3'(2'),5'-bisphosphate nucleotidase | 2.5407 | CBU_0599 | 30.4 | 4.8 | Intermediary metabolism and other metabolic pathways |
| Q83F14 | Carbonic anhydrase | 2.2036 | CBU_0139 | 23.5 | 7.2 | Intermediary metabolism and other metabolic pathways |
| Q83A78 | S-adenosylmethionine synthetase | 2.1565 | CBU_2030 | 42.7 | 5.6 | Intermediary metabolism and other metabolic pathways |
| Q83AU2 | Glucosamine--fructose-6-phosphate aminotransferase (Isomerizing) | 2.0987 | CBU_1787 | 67.3 | 9.8 | Intermediary metabolism and other metabolic pathways |
| Q83A77 | Adenosylhomocysteinase | 2.0165 | CBU_2031 | 47.8 | 6 | Intermediary metabolism and other metabolic pathways |
| Q83DV5 | Thiosulfate sulfurtransferase glpE | 1.8968 | CBU_0587 | 12.3 | 6.1 | Intermediary metabolism and other metabolic pathways |
| Q93N42 | 3'(2'),5'-bisphosphate nucleotidase | 1.7573 | CBU_0701 | 30.2 | 7.3 | Intermediary metabolism and other metabolic pathways |
| Q83DR7 | Inorganic pyrophosphatase | 1.3651 | CBU_0628 | 19.6 | 4.9 | Intermediary metabolism and other metabolic pathways |
| Q83AM7 | 3-hydroxyisobutyryl-CoA hydrolase | 6.1114 | CBU_1856 | 39.7 | 5.6 | Fatty acid and phospholipid metabolism |
| Q820W4 | 3-hydroxyacyl CoA dehydrogenase | 3.0177 | CBU_0847 | 26.9 | 8.8 | Fatty acid and phospholipid metabolism |
| Q83DW6 | Enoyl-CoA hydratase | 2.3658 | CBU_0576 | 70.7 | 9.6 | Fatty acid and phospholipid metabolism |
| Q83CX6 | Methylcrotonyl-CoA carboxylase carboxyl transferase subunit | 2.3109 | CBU_0975 | 58.7 | 7.3 | Fatty acid and phospholipid metabolism |
| Q83CX4 | Methylcrotonyl-CoA carboxylase biotin-containing subunit | 2.1048 | CBU_0977 | 74.5 | 7.9 | Fatty acid and phospholipid metabolism |
| Q83BJ8 | Acetyl-coenzyme A carboxylase carboxyl transferase subunit alpha | 2.0324 | CBU_1510 | 35.3 | 6.6 | Fatty acid and phospholipid metabolism |
| Q83B00 | Biotin carboxyl carrier protein of acetyl-CoA carboxylase | 1.9534 | CBU_1725 | 16.9 | 4.6 | Fatty acid and phospholipid metabolism |
| Q83E38 | Acyl carrier protein | 1.9335 | CBU_0496 | 92.9 | 3.7 | Fatty acid and phospholipid metabolism |
| Q83CX7 | Acetyl-CoA acetyltransferase | 1.863 | CBU_0974 | 42.2 | 7.8 | Fatty acid and phospholipid metabolism |
| Q83CX5 | Methylglutaconyl-CoA hydratase | 1.7301 | CBU_0976 | 28.2 | 6.2 | Fatty acid and phospholipid metabolism |
| Q83DW8 | 3-ketoacyl-CoA thiolase | 1.6936 | CBU_0574 | 47 | 8.4 | Fatty acid and phospholipid metabolism |
| Q820W7 | (3R)-hydroxymyristoyl-[acyl-carrier-protein] dehydratase | 1.5504 | CBU_0614 | 16.3 | 9.9 | Fatty acid and phospholipid metabolism |
| Q820W9 | 3-oxoacyl-[acyl-carrier protein] reductase | 1.4796 | CBU_0495 | 26.3 | 7.9 | Fatty acid and phospholipid metabolism |
| Q83AQ4 | Phosphatidylserine decarboxylase proenzyme | 1.3221 | CBU_1826 | 31.9 | 9.4 | Fatty acid and phospholipid metabolism |
| Q83AZ9 | Biotin carboxylase | 1.2836 | CBU_1726 | 49.2 | 6.8 | Fatty acid and phospholipid metabolism |
| Q83E40 | Fatty acid/phospholipid synthesis protein plsX | 1.2651 | CBU_0492 | 37.4 | 9.4 | Fatty acid and phospholipid metabolism |
| Q83E39 | Malonyl-CoA-[acyl-carrier-protein] transacylase | 1.1217 | CBU_0494 | 33.9 | 5.6 | Fatty acid and phospholipid metabolism |
| Q83F39 | L-threonine 3-dehydrogenase | 11.7795 | CBU_0112 | 37.9 | 7.9 | Energy metabolism-electron transport |
| Q83D15 | Glycerol-3-phosphate dehydrogenase | 5.6474 | CBU_0931 | 56 | 9.5 | Energy metabolism-electron transport |
| Q83FB4 | Ribose-5-phosphate isomerase A | 5.3328 | CBU_0026 | 23.8 | 6.5 | Energy metabolism-electron transport |
| Q83AF4 | ATP synthase epsilon chain | 4.7628 | CBU_1946 | 15.2 | 6.5 | Energy metabolism-electron transport |
| Q83AU5 | Glyceraldehyde 3-phosphate dehydrogenase | 4.4818 | CBU_1783 | 36.2 | 6.3 | Energy metabolism-electron transport |
| P18789 | Citrate synthase | 3.5865 | CBU_1410 | 48.6 | 7.6 | Energy metabolism-electron transport |
| Q83BI8 | Glutaredoxin | 3.2732 | CBU_1520 | 9.9 | 6.6 | Energy metabolism-electron transport |
| Q83C87 | Malate dehydrogenase | 2.8993 | CBU_1241 | 35.4 | 4.8 | Energy metabolism-electron transport |
| Q83CC3 | Succinate-semialdehyde dehydrogenase (NADP+) | 2.7801 | CBU_1204 | 47.9 | 6.9 | Energy metabolism-electron transport |
| Q83BH2 | 2,3-bisphosphoglycerate-independent phosphoglycerate mutase | 2.5824 | CBU_1536 | 57.5 | 5.8 | Energy metabolism-electron transport |
| Q83A24 | Thioredoxin | 2.5517 | CBU_2087 | 12.6 | 4.5 | Energy metabolism-electron transport |
| Q83DE9 | Sensor protein | 2.5022 | CBU_0789 | 90.6 | 5.4 | Energy metabolism-electron transport |
| Q83AV0 | Fructose-bisphosphate aldolase | 2.3885 | CBU_1778 | 39.5 | 10.4 | Energy metabolism-electron transport |
| Q83E67 | Dihydrolipoyl dehydrogenase | 2.3425 | CBU_0463 | 51.1 | 7.5 | Energy metabolism-electron transport |
| Q83B09 | Probable glycine dehydrogenase [decarboxylating] subunit 2 | 2.3161 | CBU_1713 | 54.6 | 8.6 | Energy metabolism-electron transport |
| Q83AF5 | ATP synthase subunit beta | 2.2366 | CBU_1945 | 50.4 | 4.7 | Energy metabolism-electron transport |
| P39916 | Thioredoxin reductase | 2.2168 | CBU_1193 | 34.6 | 6.3 | Energy metabolism-electron transport |
| P51053 | Succinate dehydrogenase iron-sulfur subunit | 2.1691 | CBU_1400 | 27.7 | 7 | Energy metabolism-electron transport |
| Q83CQ8 | Carbon storage regulator homolog 2 | 2.1256 | CBU_1050 | 7.9 | 4.4 | Energy metabolism-electron transport |
| Q83BU7 | Dihydrolipoamide succinyltransferase component (E2) of 2-oxoglutarate dehydrogenase complex | 2.0932 | CBU_1398 | 45.8 | 5.2 | Energy metabolism-electron transport |
| Q83CJ6 | Carboxymethylenebutenolidase | 2.0388 | CBU_1119 | 26.4 | 5.8 | Energy metabolism-electron transport |
| Q83AU7 | Pyruvate kinase | 2.0373 | CBU_1781 | 52.2 | 6 | Energy metabolism-electron transport |
| Q9ZH99 | Isocitrate dehydrogenase [NADP] | 2.0317 | CBU_1200 | 46.6 | 6.3 | Energy metabolism-electron transport |
| Q83BR0 | NADH-quinone oxidoreductase chain F | 2.0193 | CBU_1443 | 46.4 | 6.9 | Energy metabolism-electron transport |
| Q83AF7 | ATP synthase subunit alpha | 1.9946 | CBU_1943 | 56.8 | 5.9 | Energy metabolism-electron transport |
| Q83B44 | Enolase | 1.9888 | CBU_1674 | 46.6 | 4.4 | Energy metabolism-electron transport |
| Q83AL2 | Ribulose-phosphate 3-epimerase | 1.9403 | CBU_1872 | 24.2 | 7.8 | Energy metabolism-electron transport |
| Q83AF6 | ATP synthase gamma chain | 1.8882 | CBU_1944 | 32.6 | 8 | Energy metabolism-electron transport |
| Q83B05 | Aconitate hydratase | 1.861 | CBU_1720 | 98.3 | 5.9 | Energy metabolism-electron transport |
| Q83DW1 | Ferredoxin | 1.7833 | CBU_0581 | 12.7 | 3.8 | Energy metabolism-electron transport |
| Q83E68 | Dihydrolipoamide acetyltransferase component of pyruvate dehydrogenase complex | 1.7704 | CBU_0462 | 47.9 | 6 | Energy metabolism-electron transport |
| Q83AE4 | NAD(P) transhydrogenase subunit beta | 1.7524 | CBU_1957 | 48,8 | 9.4 | Energy metabolism-electron transport |
| P53592 | Succinyl-CoA ligase [ADP-forming] subunit beta | 1.6514 | CBU_1397 | 42.3 | 5.3 | Energy metabolism-electron transport |
| Q83BQ8 | NADH-quinone oxidoreductase subunit D | 1.6466 | CBU_1445 | 48.1 | 6.7 | Energy metabolism-electron transport |
| Q83BR1 | NADH-quinone oxidoreductase | 1.6278 | CBU_1442 | 87.6 | 5.4 | Energy metabolism-electron transport |
| Q83DG5 | Methylisocitrate lyase | 1.6269 | CBU_0771 | 31.7 | 6.1 | Energy metabolism-electron transport |
| Q83AF8 | ATP synthase subunit delta | 1.6209 | CBU_1942 | 21.1 | 10 | Energy metabolism-electron transport |
| Q83AE6 | NAD(P) transhydrogenase alpha subunit | 1.5735 | CBU_1955 | 41.3 | 9 | Energy metabolism-electron transport |
| P51054 | Succinate dehydrogenase flavoprotein subunit | 1.5263 | CBU_1401 | 65.4 | 7.1 | Energy metabolism-electron transport |
| P51056 | 2-oxoglutarate dehydrogenase E1 component | 1.4512 | CBU_1399 | 106.7 | 7.6 | Energy metabolism-electron transport |
| Q83F40 | 2-amino-3-ketobutyrate coenzyme A ligase | 1.4151 | CBU_0111 | 43.3 | 6.1 | Energy metabolism-electron transport |
| Q83EM3 | Phosphomannomutase | 1.2304 | CBU_0294 | 50.5 | 6.4 | Energy metabolism-electron transport |
| Q83DR6 | Proline dehydrogenase | 1.1937 | CBU_0629 | 116.4 | 6.7 | Energy metabolism-electron transport |
| Q83D32 | 2-methylcitrate dehydratase | 1.13 | CBU_0912 | 53.3 | 6.4 | Energy metabolism-electron transport |
| Q83E69 | Pyruvate dehydrogenase E1 component | 1.0803 | CBU_0461 | 99.7 | 6.1 | Energy metabolism-electron transport |
| Q83AU4 | Transketolase | 1.0452 | CBU_1784 | 73.6 | 5.5 | Energy metabolism-electron transport |
| Q83AF9 | ATP synthase subunit b | 0.9707 | CBU_1941 | 17.4 | 5.3 | Energy metabolism-electron transport |
| Q83CJ7 | Electron transfer flavoprotein beta-subunit | 0.7765 | CBU_1118 | 27.4 | 6.7 | Energy metabolism-electron transport |
| Q83EM6 | Ferredoxin | 0.735 | CBU_0289 | 9.3 | 4.1 | Energy metabolism-electron transport |
| Q83DM4 | Transaldolase | 0.2199 | CBU_0675 | 27.4 | 5.5 | Energy metabolism-electron transport |
| Q83DZ6 | DNA ligase | 3.7678 | CBU_0542 | 75.2 | 9.5 | DNA metabolism |
| Q83CE4 | Deoxyribodipyrimidine photolyase | 3.5402 | CBU_1176 | 55.6 | 10.1 | DNA metabolism |
| Q83C16 | Integration host factor subunit alpha | 2.7392 | CBU_1320 | 11.6 | 10.4 | DNA metabolism |
| Q83EM1 | Exodeoxyribonuclease III | 2.6992 | CBU_0297 | 30.4 | 9.3 | DNA metabolism |
| Q83AY8 | Integration host factor subunit beta | 2.6641 | CBU_1738 | 13 | 9.7 | DNA metabolism |
| Q83AL7 | Topoisomerase IV subunit A | 2.5763 | CBU_1866 | 84.3 | 9.6 | DNA metabolism |
| Q83CD5 | UvrABC system protein C | 2.5744 | CBU_1185 | 69 | 10.1 | DNA metabolism |
| Q83EL4 | ATP-dependent DNA helicase | 2.46 | CBU_0305 | 79.9 | 10.3 | DNA metabolism |
| Q83A13 | Phage integrase family protein | 2.0547 | CBU_A0010 | 47.3 | 9.9 | DNA metabolism |
| Q83C90 | Transcriptional regulator, CopG family | 1.8302 | CBU_1238 | 98.2 | 10.4 | DNA metabolism |
| Q83AA5 | DNA topoisomerase | 1.7869 | CBU_2000 | 87.2 | 8.5 | DNA metabolism |
| Q83AH2 | Probable chromosome-partitioning protein parB | 1.753 | CBU_1927 | 32 | 9.3 | DNA metabolism |
| Q83AG5 | ATP-dependent DNA ligase | 1.7486 | CBU_1934 | 89.7 | 9.9 | DNA metabolism |
| Q83FD8 | Chromosomal replication initiator protein dnaA | 1.7173 | CBU_0001 | 51 | 9.3 | DNA metabolism |
| Q83AT5 | DNA polymerase I | 1.587 | CBU_1795 | 100.9 | 9 | DNA metabolism |
| Q83E58 | Bacterial DNA-binding protein | 1.5311 | CBU_0473 | 11.6 | 11 | DNA metabolism |
| Q83EP1 | Excinuclease ABC subunit A | 1.5164 | CBU_0274 | 106.1 | 8.8 | DNA metabolism |
| Q83CQ4 | Protein recA | 1.5155 | CBU_1054 | 37.1 | 5.9 | DNA metabolism |
| Q83F84 | Topoisomerase IV subunit B | 1.4699 | CBU_0064 | 70.9 | 7 | DNA metabolism |
| Q83C37 | DNA repair protein | 1.295 | CBU_1297 | 63 | 6.4 | DNA metabolism |
| Q83E13 | DNA gyrase subunit A | 1.1808 | CBU_0524 | 94 | 8.8 | DNA metabolism |
| Q83FD5 | DNA gyrase subunit B | 1.0736 | CBU_0004 | 90.6 | 6.9 | DNA metabolism |
| Q83BN9 | DNA-binding protein HU | 1.0384 | CBU_1464 | 10.0 | 10.2 | DNA metabolism |
| Q83BT0 | DNA repair protein radA | 0.9857 | CBU_1422 | 48.7 | 7.9 | DNA metabolism |
| Q83E18 | UvrABC system protein B | 0.7362 | CBU_0518 | 77.3 | 5.4 | DNA metabolism |
| Q83C85 | Exodeoxyribonuclease 7 large subunit | 0.674 | CBU_1243 | 47.1 | 9.9 | DNA metabolism |
| Q83DN8 | DNA polymerase III subunit gamma/tau | 0.6576 | CBU_0659 | 56.8 | 6.4 | DNA metabolism |
| Q83BB7 | DNA primase | 0.6173 | CBU_1595 | 62.4 | 8.4 | DNA metabolism |
| Q83EI3 | DNA-binding protein fis | 0.5472 | CBU_0337 | 11.5 | 6.8 | DNA metabolism |
| Q83C00 | DNA polymerase III alpha subunit | 0.4357 | CBU_1337 | 128.4 | 5.8 | DNA metabolism |
| Q83AQ8 | Superoxide dismutase [Cu-Zn] | 9.1028 | CBU_1822 | 17.8 | 9.7 | Detoxification |
| Q83B14 | Thioredoxin peroxidase | 7.1352 | CBU_1706 | 21.8 | 4.8 | Detoxification |
| P19685 | Superoxide dismutase [Fe] | 7.0422 | CBU_1708 | 22.2 | 6.6 | Detoxification |
| Q83AC6 | LPS-assembly protein lptD | 1.036 | CBU_1978 | 99.0 | 9.1 | Detoxification |
| Q57333 | 27kDa outer membrane protein | 4.8345 | CBU_1910 | 27.6 | 9.5 | Cell envelope |
| Q83E02 | Putative uncharacterized protein | 4.5816 | CBU_0535 | 36 | 10.3 | Cell envelope |
| Q83D71 | Alanine racemase | 4.4175 | CBU_0869 | 39.9 | 9.2 | Cell envelope |
| Q83BN2 | Rod shape-determining protein | 3.4438 | CBU_1471 | 37.6 | 5.1 | Cell envelope |
| Q83DT1 | Outer membrane protein | 3.2839 | CBU_0612 | 18.8 | 10.4 | Cell envelope |
| Q83C68 | D-alanyl-D-alanine serine-type carboxypeptidase | 2.9479 | CBU_1261 | 46.3 | 9.7 | Cell envelope |
| Q83AY4 | Phosphoheptose isomerase | 2.7894 | CBU_1743 | 21.4 | 4.8 | Cell envelope |
| Q83DT0 | UDP-3-O-[3-hydroxymyristoyl] glucosamine N-acyltransferase | 2.6331 | CBU_0613 | 36.2 | 8.4 | Cell envelope |
| Q83BL5 | UDP-2,3-diacylglucosamine hydrolase | 2.5567 | CBU_1489 | 28.3 | 10.2 | Cell envelope |
| Q83AF3 | Bifunctional protein glmU | 2.4315 | CBU_1947 | 49.3 | 7.5 | Cell envelope |
| Q83DZ0 | Membrane-bound lytic murein transglycosylase B | 2.4061 | CBU_0548 | 38.7 | 9.9 | Cell envelope |
| Q83CA7 | Hypothetical membrane spanning protein | 2.2648 | CBU_1221 | 21.7 | 10.7 | Cell envelope |
| Q83EL2 | Outer membrane protein | 2.2066 | CBU_0307 | 24.9 | 10.2 | Cell envelope |
| Q83E52 | 3-deoxy-manno-octulosonate cytidylyltransferase | 2.1849 | CBU_0479 | 28 | 4.7 | Cell envelope |
| Q83CK4 | Membrane-bound lytic murein transglycosylase A | 2.0483 | CBU_1111 | 44.8 | 9.8 | Cell envelope |
| Q83B43 | 2-dehydro-3-deoxyphosphooctonate aldolase | 1.9601 | CBU_1675 | 30.5 | 6.3 | Cell envelope |
| Q83CW5 | Rare lipoprotein A | 1.9473 | CBU_0987 | 29.2 | 10.8 | Cell envelope |
| B5U8Q5 | Polyprenyl-phosphate beta-D-mannosyltransferase | 1.9425 | CBU_0690 | 31.6 | 9.7 | Cell envelope |
| Q83AS0 | Macrolide-specific efflux protein | 1.9282 | CBU_1810 | 42.7 | 6.8 | Cell envelope |
| Q93N54 | GDP-mannose 4,6 dehydratase | 1.8926 | CBU_0689 | 39.9 | 7.1 | Cell envelope |
| Q83DY8 | Cell elongation specific D,D-transpeptidase | 1.8181 | CBU_0550 | 69.6 | 9.5 | Cell envelope |
| Q83F27 | UDP-N-acetylmuramoyl-tripeptide--D-alanyl-D-alanine ligase | 1.6871 | CBU_0124 | 47.6 | 7.4 | Cell envelope |
| Q83F58 | Peptidoglycan-associated lipoprotein | 1.6098 | CBU_0091 | 19.8 | 9.5 | Cell envelope |
| Q83D94 | UDP-N-acetylglucosamine 4-epimerase | 1.5838 | CBU_0844 | 37.9 | 8.1 | Cell envelope |
| Q83EW0 | Outer membrane protein | 1.5812 | CBU_0198 | 65.4 | 10 | Cell envelope |
| Q83BN3 | Rod shape-determining protein | 1.53 | CBU_1470 | 32.1 | 10 | Cell envelope |
| Q83D96 | UDP-N-acetylglucosamine 2-epimerase | 1.3962 | CBU_0842 | 41.3 | 6.9 | Cell envelope |
| Q83F20 | UDP-N-acetylmuramoylalanine--D-glutamate ligase | 1.1635 | CBU_0131 | 48.7 | 7.2 | Cell envelope |
| Q83F17 | UDP-N-acetylmuramate--L-alanine ligase | 1.1608 | CBU_0136 | 50.6 | 6.5 | Cell envelope |
| Q83F28 | UDP-N-acetylmuramoyl-L-alanyl-D-glutamate--2,6-diaminopimelate ligase | 1.0646 | CBU_0123 | 53.8 | 6.8 | Cell envelope |
| Q83D93 | UDP-N-acetyl-D-galactosamine 6-dehydrogenase | 1.0631 | CBU_0845 | 47.6 | 5.4 | Cell envelope |
| P51836 | GTP-binding protein era homolog | 2.5569 | CBU_1502 | 33.8 | 10 | Cell division |
| Q83AI2 | Cell division ATP-binding protein | 2.2475 | CBU_1904 | 24.5 | 10.8 | Cell division |
| Q83AD5 | DnaK suppressor protein | 1.9216 | CBU_1969 | 16.9 | 5.1 | Cell division |
| Q83F12 | Cell division protein ftsZ | 1.4423 | CBU_0141 | 40.7 | 4.3 | Cell division |
| Q83BY5 | Cell division protein | 1.2551 | CBU_1352 | 71.6 | 6.6 | Cell division |
| P39920 | DNA translocase ftsK | 1.1958 | CBU_1191 | 85.2 | 9.1 | Cell division |
| Q81ZL2 | Chromosome partition protein | 0.8525 | CBU_0540 | 133.5 | 5.0 | Cell division |
| P24703 | Dihydrodipicolinate reductase | 8.123 | CBU_1709 | 26.2 | 7.6 | Amino acid biosynthesis |
| Q83CA6 | Dihydrodipicolinate synthase | 5.7306 | CBU_1222 | 31.6 | 5.1 | Amino acid biosynthesis |
| Q83D67 | Chorismate synthase | 4.3848 | CBU_0874 | 37.9 | 9.5 | Amino acid biosynthesis |
| Q83A36 | 3-dehydroquinate dehydratase | 4.0999 | CBU_2075 | 26.5 | 7.5 | Amino acid biosynthesis |
| Q83BY0 | Cysteine desulfurase | 3.8145 | CBU_1357 | 44.8 | 6.8 | Amino acid biosynthesis |
| Q83E11 | 3-phosphoshikimate 1-carboxyvinyltransferase | 3.1566 | CBU_0526 | 46.4 | 5.7 | Amino acid biosynthesis |
| Q83A41 | Chorismate mutase family protein | 2.8157 | CBU_2070 | 15.6 | 10.4 | Amino acid biosynthesis |
| Q83DM6 | Histidinol-phosphatase | 2.4391 | CBU_0673 | 20.5 | 8.2 | Amino acid biosynthesis |
| Q83AD4 | Diaminopimelate epimerase | 2.3949 | CBU_1970 | 30 | 6.5 | Amino acid biosynthesis |
| Q83DN1 | 2,3,4,5-tetrahydropyridine-2,6-dicarboxylate N-succinyltransferase | 2.1615 | CBU_0667 | 29.9 | 7.6 | Amino acid biosynthesis |
| Q83BT3 | Serine hydroxymethyltransferase | 2.1136 | CBU_1419 | 45.6 | 8.8 | Amino acid biosynthesis |
| Q83E19 | Aspartate aminotransferase | 1.8685 | CBU_0517 | 42.8 | 5.9 | Amino acid biosynthesis |
| Q83C54 | 4-Hydroxy-2-oxoglutarate aldolase | 1.8138 | CBU_1277 | 22.6 | 6.9 | Amino acid biosynthesis |
| Q83A84 | Cysteine synthase | 1.549 | CBU_2024 | 34.3 | 4.7 | Amino acid biosynthesis |
| Q83A83 | Cystathionine beta-lyase | 1.403 | CBU_2025 | 43.7 | 8.3 | Amino acid biosynthesis |
| Q83AJ2 | 3-dehydroquinate synthase | 1.0558 | CBU_1893 | 40.4 | 6.6 | Amino acid biosynthesis |
| Q83A62 | 5-methyltetrahydropteroyltriglutamate--homocysteine methyltransferase | 0.3972 | CBU_2048 | 88.4 | 5.7 | Amino acid biosynthesis |
| Q83D86 | Methylglyoxal synthase | 2.8622 | CBU_0853 | 16.5 | 6.2 | Adaptations to atypical conditions-stress response |
| Q83C56 | Starvation sensing protein | 2.4883 | CBU_1275 | 45.4 | 6 | Adaptations to atypical conditions-stress response |
| Q83AC1 | Universal stress protein A homolog 1 | 2.4498 | CBU_1983 | 15.7 | 6.4 | Adaptations to atypical conditions-stress response |
| Q83BW2 | GTP pyrophosphokinase | 1.5423 | CBU_1375 | 81.4 | 8 | Adaptations to atypical conditions-stress response |
| Q83AY0 | Stringent starvation protein A homolog | 0.8009 | CBU_1747 | 24.4 | 5.6 | Adaptations to atypical conditions-stress response |
| Q83DW0 | BolA | 0.7473 | CBU_0582 | 8.8 | 5.8 | Adaptations to atypical conditions-stress response |

All 531 proteins were identified in both samples (i. e. *C. burnetii* cultured in presence of tetracycline and *C. burnetii* cultured with no antibiotic present) in different expression levels. Proteins are categorized according to their predicted cellular function; their ratio value, gene locus, molecular weight (MW), isoelectric point and predicted cellular function are indicated.
