# Supplementary material for: Quantitative Proteome Profiling of C. burnetii under Tetracycline Stress Conditions
Source: PLoS One. 2012 Mar 16;7(3):e33599. doi: 10.1371/journal.pone.0033599 (PMC3306420; doi:10.1371/journal.pone.0033599)
Supplement: Table S2 — Proteins that were significantly over-expressed in C. burnetii when cultured in presence of tetracycline (Q212Dox) or in C. burnetii when cultured with no antibiotic (Q212). (DOCX) [file pone.0033599.s002.docx]

**Table S2:** Proteins that were significantly over-expressed in *C. burnetii* when cultured in presence of tetracycline (Q212Dox) or in *C. burnetii* when cultured with no antibiotic (Q212).

| **Gene locus (JCVI-CMR)** | **Accession no. (Uniprot KB)** | **Description Uniprot (Uniprot KB)** | **L/H Ratio** | **Strain over-expressed** | **MW (ProtParam)** | **Theoretical pI (ProtParam)** | **GRAVY Score (ProtParam)** | **Subcellular Localization Prediction (PSORTb v.3.0)** | **Cellular Role Category (JCVI-CMR)** |
| --- | --- | --- | --- | --- | --- | --- | --- | --- | --- |
| CBU_1169 | Q83CE9 | Small heat shock protein (Hsp 20 family) | 118.92 | Q212 | 17.2 | 5.6 | -0.683 | Unknown | Protein fate: Protein folding and stabilization |
| CBU_1550 | **Q83BF9** | Phosphoenolpyruvate-protein phosphotransferase (ptsP) | 70.15 | Q212 | 84.9 | 5.1 | -0.056 | Cytoplasmic | Transport and binding proteins: Carbohydrates, organic alcohols, and acids |
| CBU_1095 | Q83CL9 | Hypothetical exported protein | 47.65 | Q212 | 20.2 | 6.8 | -0.047 | Unknown | Unknown |
| CBU_1677 | Q83B41 | Hypothetical cytosolic protein | 25.52 | Q212 | 17.3 | 6.2 | -0.967 | Unknown | Unknown |
| CBU_0632 | Q83DR4 | Putative uncharacterized protein | 21.23 | Q212 | 11.8 | 4.6 | -0.917 | Unknown | Unknown |
| CBU_0943 | Q83D04 | Rhodanese-related sulfurtransferase | 21.15 | Q212 | 16.5 | 8.6 | -0.452 | Unknown | Unknown |
| CBU_2029 | Q83A79 | Hypothetical exported protein | 20.55 | Q212 | 24.4 | 10.1 | -0.706 | Unknown | Unknown |
| CBU_0016 | Q83FC4 | Xanthosine phosphorylase | 20.35 | Q212 | 30.1 | 6.8 | -0.003 | Unknown | Purines, pyrimidines, nucleosides, and nucleotides: Salvage of nucleosides and nucleotides |
| CBU_0744 | Q820W5 | Hpr(Ser) kinase | 12.77 | Q212 | 26.4 | 6.9 | -0.164 | Unknown | Transport and binding proteins: Carbohydrates, organic alcohols, and acids |
| CBU_1766 | Q83AW2 | Ferrous iron transport protein B | 12.63 | Q212 | 89.9 | 10 | 0.286 | Cytoplasmic membrane | Transport and binding proteins: Cations and iron carrying compounds |
| CBU_0321 | Q83EJ9 | IcmH | 12.14 | Q212 | 29.1 | 6 | -0.122 | unknown | unknown |
| CBU_0112 | Q83F39 | L-threonine 3-dehydrogenase | 11.77 | Q212 | 37.9 | 7.9 | 0.052 | Cytoplasmic | Energy metabolism: Amino acids and amines |
| CBU_0425 | Q83EA1 | Putative uncharacterized protein | 10.44 | Q212 | 51.8 | 5.7 | -0.507 | unknown | unknown |
| CBU_2010 | Q83A96 | Hypothetical exported protein | 10.24 | Q212 | 12 | 10.6 | -0.615 | Cytoplasmic membrane | unknown |
| CBU_1267.1 | B5QSC0 | ScvA | 9.34 | Q212 | 3.6 | 12.1 | -2.583 | unknown | unknown |
| CBU_1822 | Q83AQ8 | Superoxide dismutase [Cu-Zn] | 9.10 | Q212 | 17.8 | 9.7 | 0.049 | Periplasmic | Detoxification |
| CBU_1280.1 | B5QSC3 | Putative uncharacterized protein | 9.07 | Q212 | 8.8 | 9.8 | -0.339 | unknown | unknown |
| CBU_1709 | P24703 | Dihydrodipicolinate reductase | 8.12 | Q212 | 26.2 | 7.6 | -0.097 | Cytoplasmic | Amino acid biosynthesis: Aspartate family |
| CBU_0743 | [Q83DI7](http://www.uniprot.org/uniprot/Q83DI7) | Phosphocarrier protein HPr | 8.10 | Q212 | 10 | 8.4 | -0.274 | Cytoplasmic | Transport and binding proteins: Carbohydrates, organic alcohols, and acids |
| CBU_1099 | Q83CL5 | Signal peptidase I | 0.11 | Q212Dox | 30.1 | 10.1 | -0.119 | Cytoplasmic membrane | Protein fate: Protein and peptide secretion and trafficking |
| CBU_0675 | Q83DM4 | Transaldolase | 0.2199 | Q212Dox | 27.4 | 5.5 | -0.114 | Unknown | Energy metabolism: Pentose phosphate pathway |
| CBU_0424 | Q83EA2 | 3-methyl-2-oxobutanoate hydroxymethyltransferase | 0.37 | Q212Dox | 28.9 | 7.5 | 0.048 | Unknown | Biosynthesis of cofactors, prosthetic groups, and carriers: Pantothenate and coenzyme A |
| CBU_2048 | Q83A62 | 5-methyltetrahydropteroyltriglutamate--homocysteine methyltransferase | 0.39 | Q212Dox | 88.4 | 5.7 | -0.270 | Unknown | Amino acid biosynthesis: Aspartate family |
| CBU_1337 | Q83C00 | DNA polymerase III alpha subunit | 0.4357 | Q212Dox | 128.4 | 5.8 | -0.120 | Cytoplasmic | DNA metabolism: DNA replication, recombination, and repair |
